# Phosphatidyl Inositol 3 Kinase-Gamma Balances Antiviral and Inflammatory Responses During Influenza A H1N1 Infection: From Murine Model to Genetic Association in Patients

**DOI:** 10.3389/fimmu.2018.00975

**Published:** 2018-05-15

**Authors:** Cristiana C. Garcia, Luciana P. Tavares, Ana Carolina F. Dias, Fernanda Kehdy, Lucia Elena Alvarado-Arnez, Celso M. Queiroz-Junior, Izabela Galvão, Braulio H. Lima, Aline R. Matos, Ana Paula F. Gonçalves, Frederico M. Soriani, Milton O. Moraes, João T. Marques, Marilda M. Siqueira, Alexandre M. V. Machado, Lirlândia P. Sousa, Remo C. Russo, Mauro M. Teixeira

**Affiliations:** ^1^Laboratório de Vírus Respiratórios e do Sarampo, Instituto Oswaldo Cruz, Fundação Oswaldo Cruz (Fiocruz), Rio de Janeiro, Brazil; ^2^Laboratório de Imunofarmacologia, Departamento de Bioquímica e Imunologia, Instituto de Ciências Biológicas, Universidade Federal de Minas Gerais, Belo Horizonte, Brazil; ^3^Laboratório de Imunologia e Mecânica Pulmonar, Departamento de Fisiologia e Biofísica, Instituto de Ciências Biológicas, Universidade Federal de Minas Gerais, Belo Horizonte, Brazil; ^4^Laboratório de Hanseníase, Instituto Oswaldo Cruz, Fundação Oswaldo Cruz (Fiocruz), Rio de Janeiro, Brazil; ^5^Coordinación Nacional de Investigación, UNIFRANZ, La Paz, Bolivia; ^6^Departamento de Morfologia, Instituto de Ciências Biológicas, Universidade Federal de Minas Gerais, Belo Horizonte, Brazil; ^7^Laboratório de Inflamação e Dor, Departamento de Farmacologia, Prédio Central, Universidade de São Paulo, Ribeirão Preto, Brazil; ^8^Laboratório de Imunologia de Doenças Virais, Centro de Pesquisas René Rachou, Fundação Oswaldo Cruz (Fiocruz), Belo Horizonte, Brazil; ^9^Departamento de Biologia Geral, Instituto de Ciências Biológicas, Universidade Federal de Minas Gerais, Belo Horizonte, Brazil; ^10^Laboratório de RNA de Interferência, Departamento de Bioquímica e Imunologia, Instituto de Ciências Biológicas, Universidade Federal de Minas Gerais, Belo Horizonte, Brazil; ^11^Departamento de Análises Clínicas e Toxicológicas, Faculdade de Farmácia, Universidade Federal de Minas Gerais, Belo Horizonte, Brazil

**Keywords:** neutrophils, CD8^+^ T cells, natural killer cells, type-I IFN, p38, disease severity, single-nucleotide polymorphism

## Abstract

Influenza A virus (IAV) infection causes severe pulmonary disease characterized by intense leukocyte infiltration. Phosphoinositide-3 kinases (PI3Ks) are central signaling enzymes, involved in cell growth, survival, and migration. Class IB PI3K or phosphatidyl inositol 3 kinase-gamma (PI3Kγ), mainly expressed by leukocytes, is involved in cell migration during inflammation. Here, we investigated the contribution of PI3Kγ for the inflammatory and antiviral responses to IAV. PI3Kγ knockout (KO) mice were highly susceptible to lethality following infection with influenza A/WSN/33 H1N1. In the early time points of infection, infiltration of neutrophils was higher than WT mice whereas type-I and type-III IFN expression and p38 activation were reduced in PI3Kγ KO mice resulting in higher viral loads when compared with WT mice. Blockade of p38 in WT macrophages infected with IAV reduced levels of interferon-stimulated gene 15 protein to those induced in PI3Kγ KO macrophages, suggesting that p38 is downstream of antiviral responses mediated by PI3Kγ. PI3Kγ KO-derived fibroblasts or macrophages showed reduced type-I IFN transcription and altered pro-inflammatory cytokines suggesting a cell autonomous imbalance between inflammatory and antiviral responses. Seven days after IAV infection, there were reduced infiltration of natural killer cells and CD8^+^ T lymphocytes, increased concentration of inflammatory cytokines in bronchoalveolar fluid, reduced numbers of resolving macrophages, and IL-10 levels in PI3Kγ KO. This imbalanced environment in PI3Kγ KO-infected mice culminated in enhanced lung neutrophil infiltration, reactive oxygen species release, and lung damage that together with the increased viral loads, contributed to higher mortality in PI3Kγ KO mice compared with WT mice. In humans, we tested the genetic association of disease severity in influenza A/H1N1pdm09-infected patients with three potentially functional *PIK3CG* single-nucleotide polymorphisms (SNPs), rs1129293, rs17847825, and rs2230460. We observed that SNPs rs17847825 and rs2230460 (A and T alleles, respectively) were significantly associated with protection from severe disease using the recessive model in patients infected with influenza A(H1N1)pdm09. Altogether, our results suggest that PI3Kγ is crucial in balancing antiviral and inflammatory responses to IAV infection.

## Introduction

Influenza A and B viruses are among the most common causes of acute respiratory viral diseases and account for 3–5 million cases of severe infection and 290,000–650,000 deaths worldwide every year (1). Influenza A and B are negative sense, eight-segmented single-stranded RNA virus belonging to the *Orthomyxoviridae* family (2).

The disease caused by highly pathogenic influenza A virus (IAV) strains is often related to an intense or uncontrolled inflammatory response that causes serious lung damage and severe clinical manifestations (3–5). The immune response against the virus is first mediated by type-I and type-III IFNs, mainly produced by epithelial cells, which are the primary target of IAV infection, and by dendritic cells (6). Natural killer (NK) cells, macrophages, and neutrophils are the first cell types actively recruited by the action of cytokines and chemokines to the lungs and airways in response to the virus infection (7). Later, specific CD8^+^ T cells arrive at the site of infection and actively kill infected cells (8). Whereas the activation and recruitment of leukocytes is important to control infection, excessive activation of neutrophils and macrophages, might be harmful to the host (9, 10). Different genetic polymorphisms on host factors genes of recognition, signaling or activating molecules have been investigated to explain the diversity of immune responses to influenza infection and how this might affect disease outcome (11–15).

The signaling of many inflammatory mediators like chemokines, complement fragments, and phospholipids is mediated by its binding to a G-protein-coupled receptor (GPCR) specific for each ligand (16). The binding to GPCRs triggers diverse intracellular signaling events, including activation of the phosphatidyl inositol 3 kinase-gamma (PI3Kγ), followed by AKT activation and downstream pathways that promote cell survival, migration, and proliferation (17). PI3Kγ, the only member of class IB phosphoinositide-3 kinase (PI3K), is expressed by neutrophils, eosinophils, macrophages, T cells and mast cells, endothelial cells, fibroblasts, and cardiomyocytes (18). The other PI3K isoforms (class IA PI3K), PI3Kα, PI3Kβ, and PI3Kδ are activated by tyrosine-kinase coupled receptors (19). PI3Kβ can also be activated directly by GPCR activation (20). It has already been reported that the protein NS1 of IAV can activate class IA PI3Ks, which contribute to viral particles entrance into host cells (21) and cell survival (22). PI3K activation during IAV infection is dependent on TLR3 activation and causes enhanced expression of the antiviral cytokine IFN-β and the chemokines CXCL8 and CCL5 (23), which might be related to the recruitment of leukocytes to the site of infection. Recently, the involvement of PI3Kγ in regulating the resident dendritic cell priming of CD8^+^ T cells during influenza A infection which causes enhanced susceptibility of PI3Kγ knockout (KO) to influenza infection was described (24).

Due to the importance of PI3Kγ for the recruitment and survival of macrophages and neutrophils, which might cause lung damage when excessively activated during IAV infection, we aimed to investigate the role of PI3Kγ in the innate immunity and inflammatory responses during IAV infection. Here we describe, for the first time, that PI3Kγ is essential for the innate immune responses against IAV and resolution of inflammation triggered by the infection. Altogether, PI3Kγ plays a crucial role in driving type-I and type-III IFN production and recruitment of NK and CD8^+^ T cells, therefore controlling viral titers in lungs of infected mice. Importantly, we showed also for the first time that the single-nucleotide polymorphisms (SNPs) rs17847825 and rs2230460 located in *PIK3CG* gene are associated with disease protection in influenza A(H1N1)pdm09-infected patients.

## Materials and Methods

### Mice

C57BL/6J male mice, 8- to 10-week old, from the Central Animal Facilities of Universidade Federal de Minas Gerais (CEBIO-UFMG), PI3Kγ KO mice from the Immunopharmacology Lab (Universidade Federal de Minas Gerais/Brazil) and mice with a targeted mutation causing loss of kinase activity of PI3Kγ, here called PI3Kγ KD/KD from Centro de Criação de Camundongos Especiais (FMRP/USP, Brazil), were kept under pathogen free conditions with food and water *ad libitum*.

### Virus Strain, Cell Line, and Plaque Assay

Influenza A/H1N1/WSN/33 (herein called WSN) was propagated in Madin–Darby Canine Kidney (MDCK) cells (25, 26). Quantification of influenza virus stocks, particles in lungs, fibroblasts, or bone marrow-derived macrophages (BMDM) supernatants was performed using the plaque assay under MDCK cell monolayer, as previously described (25).

### Mice Infections and Samples Harvesting

WT, PI3Kγ KO, and PI3Kγ KD/KD mice received 25 µl of 10^4^ plaque-forming units (PFU) of WSN virus or sterile phosphate-buffered saline (PBS) *via* intranasal route under light anesthesia. For survival experiments, body weight and lethality were assessed daily until 21 days after infection. Evaluation of cellular infiltration, cytokine production, viral load, or histology analysis was performed after 3, 5, and 7 days of IAV infection. To this aim, mice were killed with an overdose of ketamine/xylazine (180 and 20 mg/kg, *via* intraperitoneal injection); bronchoalveolar lavage (BAL) was harvested by inserting and recovering for three times two aliquots of 1 mL of PBS (25), and the lungs were perfused with PBS to remove circulating blood. Left lobe was collected for histology, upper right lobe for viral quantification, medium right lobe for Western blot (WB) and real-time PCR, and two lower right lobes were harvested for myeloperoxidase (MPO) and cytokines measurements. Specific leukocyte populations in lungs and airways and activation of lung leukocytes were evaluated 7 days after infection by flow cytometry and chemoluminescence assays, respectively.

### Differentiation of Fibroblasts and BMDM, *In Vitro* Treatment, and Infection

Lung-derived fibroblasts were obtained from WT and PI3Kγ KO mice. Lungs were excised and cut into 1 mm pieces and cultured with Dulbecco’s Modified Eagle’s Medium (DMEM) containing 10% fetal bovine serum (FBS; Gibco, Carlsbad, CA, USA) in 6-well plates. Past 10 days, tissue pieces were removed, and cells were cultured for another 10 days. Then, fibroblasts were trypsinized and plated in a density of 1 × 10^4^ cells/well on 48-well plates and infected after 48 h with WSN using a multiplicity of infection (MOI) of 3.0 in DMEM with 10% FBS. After adsorption, cells were washed with DMEM to remove unbound virus. Cells were cultured for 24 h and were frozen for gene expression analysis by real-time PCR. Supernatants were collected for virus titer and cytokines measurements. Macrophages were derived from WT and PI3Kγ KO mice bone marrow. Cells were cultured in 6-well plates in DMEM containing 10% FBS and 20% L929 cell-conditioned medium as a source of macrophage colony-stimulating factor, at 37°C in a 5% CO_2_ atmosphere. On the seventh day, when the cells were completely differentiated into macrophages, they were trypsinized and seeded on 24-well plates (5 × 10^5^ cells/well). After overnight incubation, cells were infected with WSN virus as previously described for fibroblasts infection. In a first experiment, after 1 h of WSN infection, washed cells were frozen completely dry to perform WB analysis. In separate plates, supernatants were collected 24 h after infection for virus titer and cytokine quantifications and cells were frozen for gene expression analysis by real-time PCR. In another set of experiments WT BMDM were pre-treated with 5 µM of the p38 inhibitor SB 203580 (Sigma-Aldrich, St. Louis, MO, USA) diluted in dimethyl sulfoxide 0.036% in DMEM or vehicle (WT and PI3Kγ KO cells) for 1 h, then cells were mock infected or infected with WSN (MOI of 3.0) in the presence or absence of SB 203580. After 6 h, cells were frozen for further interferon-stimulated gene 15 (ISG15) protein production analyses by WB.

### Assessment of Lung MPO Levels

Lung tissue (50 µg) was homogenized in protease inhibitors cocktail and further used in ELISA and MPO assay, as previously described (27).

### ELISA

Supernatant of bronchoalveolar lavage fluid (BALF), lung homogenates, and BMDM supernatants were used to assess the concentration of cytokines IL-6, IL-10, TNF-α, and chemokine CXCL1 levels by ELISA assay, according to the manufacturer’s instructions (R&D Systems, Minneapolis, MN, USA).

### Flow Cytometry Analysis

At day 7 after IAV infection, BAL was performed and the recovered cells were used for antibody staining for FACS analysis after erythrocyte isotonic lyses. Lung leukocytes were extracted after digestion of tissue with 200 U/mL of collagenase (Sigma) and 50 U/ml of DNase I (Sigma). Cells were stained for the following surface markers: B220 (FITC, clone RA3-6B2, dilution 1:20), CD3 (PeCy7, clone 17A2, dilution 1:50), CD4 (APC Cy 7, clone GK1.5, dilution 1:100), CD8 (PercP: 53-6.7 1:100), CD11b (FITC or PECy5, clones M1/70, dilution 1:100), CD19 (Alexa647, clone 6D5, dilution 1:200), CD45 (PECy5, clone 30-F11, dilution 1:200), F4/80 (PeCy7, clone BM8, dilution 1:100), Gr1 (APC or FITC, clones RB68C5, dilution 1:100), and NK1.1 (PE, clone PK136, dilution 1:200). Stained cells were acquired by the cytometer FACSCantoII and analyzed using the FlowJo software (TreeStar, Ashland, OR, USA). Gating strategies for lymphocytes (1), NK and B cells (2), macrophages and neutrophils in BALF (3), and macrophages and neutrophils in lungs (4) are presented in Figure S1 in Supplementary Material.

### Chemiluminescence Assay

Lung leukocytes were plated at density of 1 × 10^6^ cells (WT- and KO-infected mice and WT Mock) and 0.6 × 10^6^ (KO Mock) in an NUNC FluorNunc MaxiSorp Surface 96-well plate (NUNC, Rochester, NY, USA) with Luminol (0.05 mM, Sigma) with 1 × 10^7^ particles of Zymosan A (Sigma-Aldrich) or medium. Zymosan induced luminescence was measured in a luminometer (Packard, Waltham, MA, USA) for 120 min, and the results were expressed as relative luminescence units compared with controls without Zymosan. Area under the curve was measured and compared among the groups.

### WB Analysis

Cell extracts were obtained from lung homogenates or BMDM (three to five per infected groups, one to two for mock groups), and 40 µg of whole extract was electrophoresed in 10 or 15% polyacrylamide gel electrophoresis for detection of ISG15, phospho-AKT, phospho-p38, phospho-STAT-1, and β-actin as previously described (28). Levels of proteins were normalized to the levels of β-actin in the same sample and quantified by densitometry using the ImageJ software (NIH, Bethesda, MD, USA).

### Histology Analysis

Left lobes of lungs from Mock or IAV-infected mice were fixed in formalin, dehydrated in ethanol and then embedded in paraffin to be further cut in 4 µm sections for hematoxylin and eosin (H&E) staining. Each sample was examined in different sections by a pathologist without previous knowledge about the experimental groups. A score system of 23 points was performed, as previously described (29) to evaluate airway, vascular, parenchymal inflammation, epithelial injury, and polymorphonuclear (PMN) infiltration.

### Real-Time PCR

Total RNA was extracted from lung tissue (50–100 mg) using Trizol/chloroform protocol as described by the manufacturer and from BMDM and fibroblasts using RNeasy mini kit (Qiagen, Hilden, Germany). Preparation of cDNA was performed using SuperScript™ III Reverse Transcriptase kit (Invitrogen, Life technologies, Carlsbad, CA, USA), according to the manufacturer’s instructions. Real-time PCR was performed using Power SYBRGreen PCR Master Mix 2X (Applied Biosystems, Foster City, CA, USA) on a Step One PCR System (Applied Biosystems, Foster City, CA, USA). Relative expression of *ifn-*β, *ifn-*α*4*, and *ifn-*λ*2/3* was determined by 2^−ΔCt^ (30), compared with the constitutive genes glyceraldehyde 3-phosphate dehydrogenase for *in vitro* experiments or Ribosomal protein L4 for *in vivo* experiments. The choice of the reference genes was made after a qPCR amplification of representative samples (*n* = 2–3 for each experimental group) with four reference gene primer sets. The results were analyzed using the web-based comprehensive tool (RefFinder), to compare and select the best reference gene for the experimental models/conditions based on the lowest value of the algorithms that indicates the most stable gene for each condition.

### Statistical Analysis From *In Vitro* and *In Vivo* Mice Studies

Analysis and graphs were performed in GraphPad Prism 4.0. Data are presented as the mean ± SD. Data were tested for normality using the Bartlett’s test for equal variances. As the data were normally distributed, the comparison among more than two groups was made by one-way ANOVA, followed by Newman–Keuls post-test to compare all groups. Two groups were compared by unpaired *t*-test; survival curves were compared by Log-rank test. Area under the curve was measured for each sample and total values analyzed using one-way ANOVA, followed by Newman–Keuls. Results were considered significantly different when *p* < 0.05.

### Genetic Association Study

#### Selection of Human Samples

The Laboratory of Respiratory Viruses and Measles as the National Influenza Center from WHO and from the Brazilian Influenza Surveillance System receives nasopharyngeal swabs samples or post mortem specimens from influenza suspect cases. Influenza A(H1N1)pdm09 positive samples were classified according to the clinical symptoms as influenza-like illness (ILI, *n* = 120), severe acute respiratory infection (SARI, *n* = 137), or deceased (*n* = 92). The inclusion criterion for ILI was the presence of fever and/or cough; exclusion criteria were: being a deceased patient, patient in intensive care unit, or in hospital admission, or presenting one of following symptoms: respiratory distress, O_2_ saturation lower than 95%, pneumonia. For SARI patients, the inclusion criteria were fever and/or cough and dyspnea or patients with respiratory distress with O_2_ saturation lower than 95%.

#### Selection of SNPs on *PIK3CG*

From the 85 potentially functional SNPs (located in the coding region) on the *PIK3CG* gene, we selected all variants that are available in the EPIGEN-Brazil Project database (31) that had minor allele frequency (MAF) between 8 and 25% in three Brazilian cohorts. This criterion was used to guarantee statistical potential in genetic association analyzes. Three SNPs were selected (rs1129293, rs17847825, and rs2230460), and the information regarding them are detailed in Table 1.

**Table 1:** PIK3CG SNPs selected to the study and allele frequencies (AFs) found by genetic studies with different populations.

Selected *PIK3CG* SNPs (chromosome 7)							Allele	EPIGEN-Brazil Project MAF				l000Genomes Project (Phase 3)			
								Total (*n =* 5,823)	Brazilian cohorts			Global (*n =* 1,354)	Continental groups		
rs dbSNP ID	Function	Amino acids	mRNA mutation	Codons	SIFT	PolyPhen			Bambui (*n =* 926)	Pelotas (*n =* 3,651)	Salvador (*n =* 1,246)		AMR^a^ (*n =* 347)	AFR^a^ (*n =* 504)	EUR^a^ (*n =* 503)
rsl7847825	Missense	**S/Y**	S1635Y	T**C**C/T**A**C	Deleterious (0.01)	Possibly damaging (0.873)	**A**	0.086	0.080	0.097	0.061	0.055	0.048	0.005	0.110
rs1 129293	Synonymous	**S**	S2335S	AG**C**/AG**T**			**T**	0.240	0.240	0.258	0.190	0.252	0.428	0.067	0.314
rs2230460	Synonymous	**D**	D2850D	GA**C**/GA**T**			**T**	0.088	0.082	0.098	0.065	0.059	0.050	0.011	0.112

*MAF, minor allele frequency; AFR, African populations (ESN, GWD, LWK, MSL, and YRI); AMR, native American admixed populations (CLM, MXL, PEL, and PUR); EUR, European populations (CEU, FIN, GBR, IBS, and TSI); PolyPhen, polymorphism phenotyping; SIFT, sorting intolerant from tolerant; SNP, single-nucleotide polymorphism*.

*^a^Public data from 1000 Genomes Project (32)*.

*Nucleotide changes are highlighted in bold*.

#### SNP Genotyping and Analysis

DNA was extracted from clinical samples using the PureLink™ Genomic DNA Mini Kit (Invitrogen, USA) according to the manufacturer’s instructions. The SNPs were genotyped using made to order TaqMan SNP Genotyping Assays (Invitrogen, USA): C__16179129_10, C__25472003_20, and C__34035502_10. Epidemiological variables (total number and frequency) of the recruited patients were stratified between the three clinical groups: ILI, SARI and deceased. The comparison between each group was performed with Kruskal–Wallis or chi-square tests when appropriate. Unconditional logistic regression was used to determine if the genotyped polymorphisms had an influence over the outcome. In a first round of analysis, there was no covariate inclusion in the regression model. Later non-genetic covariates were added to the model: sex, age (0–18, 19–65, and >65 years), both considered as categorical variables. The codominant, dominant and recessive models were tested comparing: ILI with each of the severe disease forms (a) versus SARI or (b) versus deceased and (c) versus the combination of SARI and deceased patients. Finally, to determine if the SNPs were involved with progression toward fatal outcome, (d) SARI group was compared against deceased group. We performed descriptive and genetic analysis using R environment version 3.1.3 with the packages epiDisplay, genetics and SNPassoc. Statistical significance was considered with *p*-values < 0.05.

## Results

### PI3Kγ KO and KD/KD Mice Succumb to Influenza A Infection

**Figure 1:**
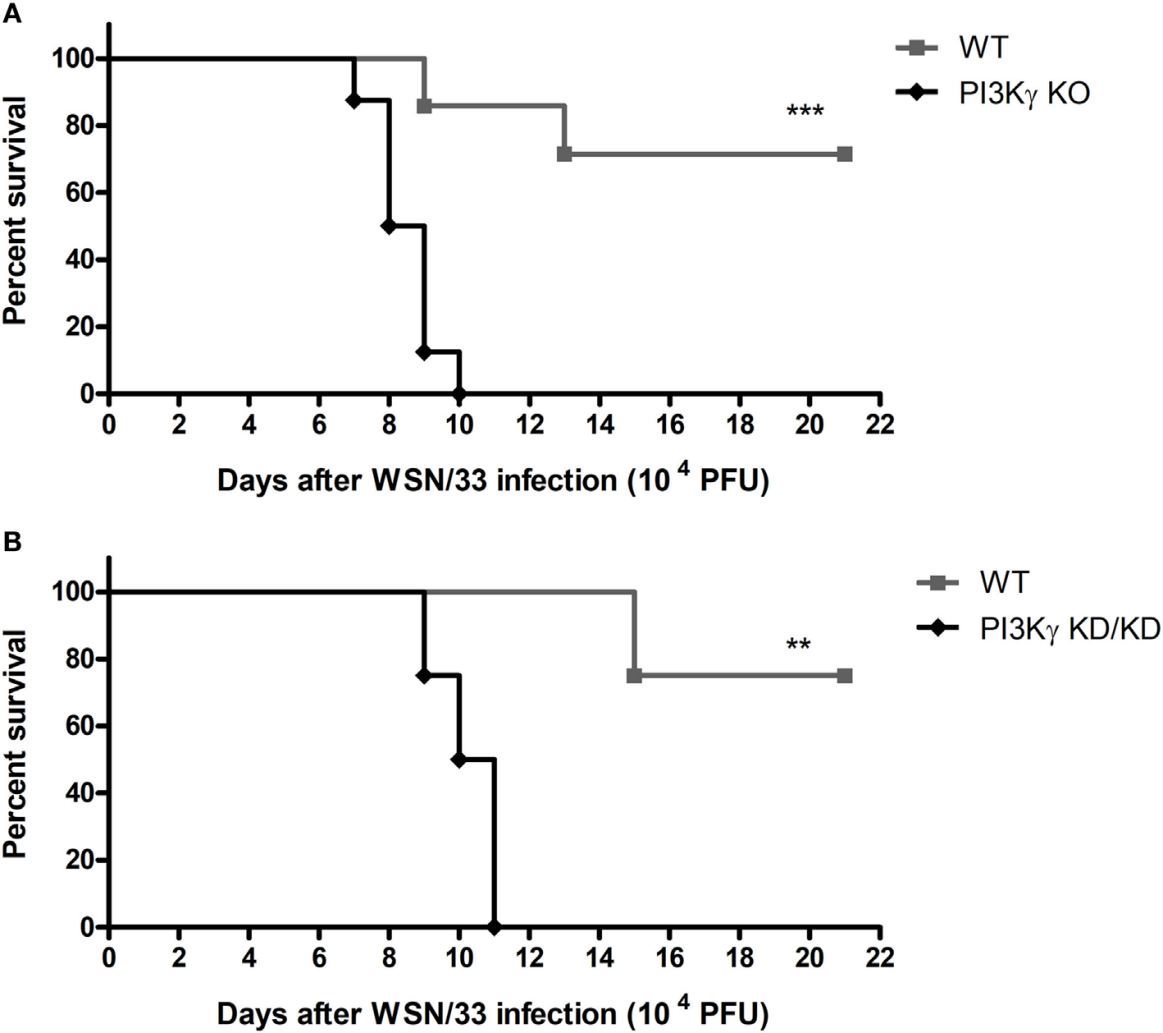
Lethality after influenza A virus infection in WT, phosphatidyl inositol 3 kinase-gamma (PI3Kγ) knockout (KO), and PI3Kγ KD/KD mice. **(A)** Lethality of WT and PI3Kγ KO mice intranasally infected with 10^4^ plaque-forming units (PFU) of WSN; result is representative of two independent experiments with seven to eight mice per group (Log-rank test; ***p < 0.001). **(B)** Lethality of WT and PI3Kγ KD/KD mice intranasally infected with 10^4^ PFU of WSN; result is representative of two independent experiments with four to eight mice per group (Log-rank test; **p < 0.01).

Initially we evaluated if the absence of PI3Kγ could affect mice survival to the mouse-adapted virus influenza A/WSN/33 H1N1 (WSN) infection. Thus, we infected WT and PI3Kγ KO mice with 10^4^ PFU of WSN. In WT mice, infection caused 28.5% of lethality (Figure 1A). In contrast, all PI3Kγ KO mice died up to day 10 after infection (Figure 1A). To investigate if the kinase activity of PI3Kγ is important to the observed phenotype, mice carrying a targeted mutation on the kinase domain of PI3Kγ, here called as PI3Kγ KD/KD, were infected with WSN. In a similar way to KO mice, all PI3Kγ KD/KD mice died after 11 days of infection whereas 75% of WT mice survived (Figure 1B).

### IAV Induces Higher Neutrophilic Infiltration in PI3Kγ KO Compared With WT Mice in the First Days of Infection

**Figure 2:**
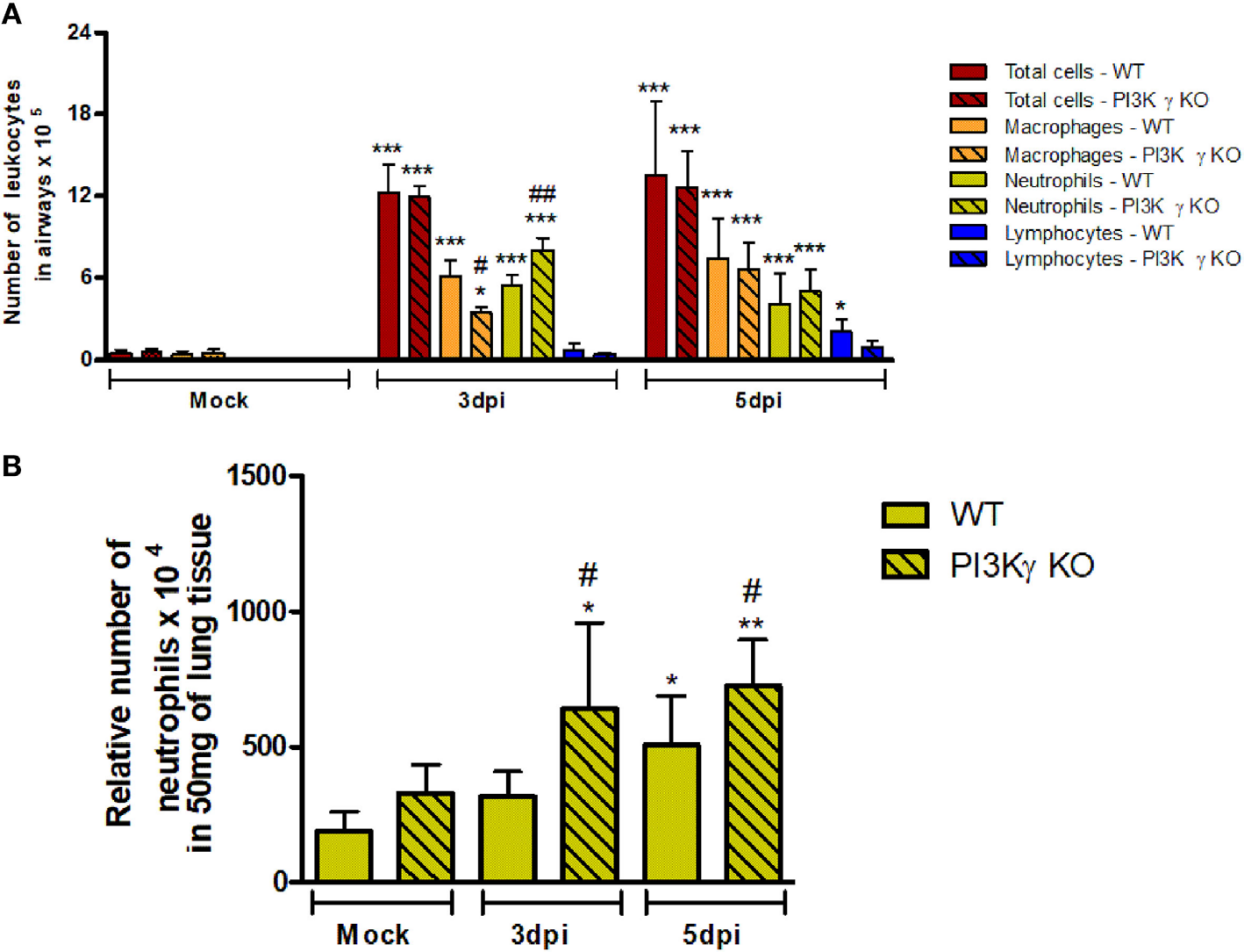
Early leukocyte recruitment to the airways and neutrophil recruitment to the lungs during influenza A virus infection are more pronounced in phosphatidyl inositol 3 kinase-gamma (PI3Kγ) knockout (KO) mice. WT and PI3Kγ KO mice were infected with WSN or phosphate-buffered saline (Mock control) and killed at days 3 and 5 after infection (*n* = 4–8 mice per group and time point). **(A)** Bronchoalveolar lavage was performed to assess recruitment of total leukocytes (red columns), macrophages (orange columns), neutrophils (yellow columns), and lymphocytes (blue columns) to the airways in cytospin stained slides. **(B)** Recruitment of neutrophils to the lungs was assessed by myeloperoxidase assay. Cell numbers are presented as mean ± SD. *p < 0.05, **p < 0.01, and ***p < 0.001, comparing to Mock or indicated groups and ^#^*p* < 0.05, ^##^*p* < 0.01, and ^###^*p* < 0.001, when compared with WT control in the same time point (one-way ANOVA, Newman–Keuls).

To investigate the causes of the increased susceptibility of PI3Kγ KO mice to IAV infection, we evaluated cellular infiltration into airways and lungs at 3 and 5 days after infection. Total leukocyte, macrophages, neutrophils and lymphocytes recruitment was assessed in BALF (Figure 2A). Total leukocytes were increased in airways of WT mice and similarly in PI3Kγ KO mice at the analyzed time points after infection. Neutrophils were recruited into the airways of WT and PI3Kγ KO mice early during infection, peaking at day 3 and reducing thereafter. Remarkably, recruitment of neutrophils into the airways of PI3Kγ KO mice was more intense than in WT mice at day 3 after infection. The influx of macrophages was elevated throughout the observed period. In comparison with WT mice, the recruitment of macrophages was reduced at day 3 after infection in PI3Kγ KO mice. Lymphocytes were found in increased numbers after IAV infection in WT but not PI3Kγ KO mice as early as 5 days post infection. By measuring the levels of the enzyme MPO, we found a significant accumulation of neutrophils in lung parenchyma of WT mice at day 5 after infection (Figure 2B), whereas in PI3Kγ deficient mice, levels of MPO were already augmented at day 3 after infection and were higher than those found in WT mice throughout the course of infection (Figure 2B).

### Impaired Early Antiviral Response in the Absence of PI3Kγ

**Figure 3:**
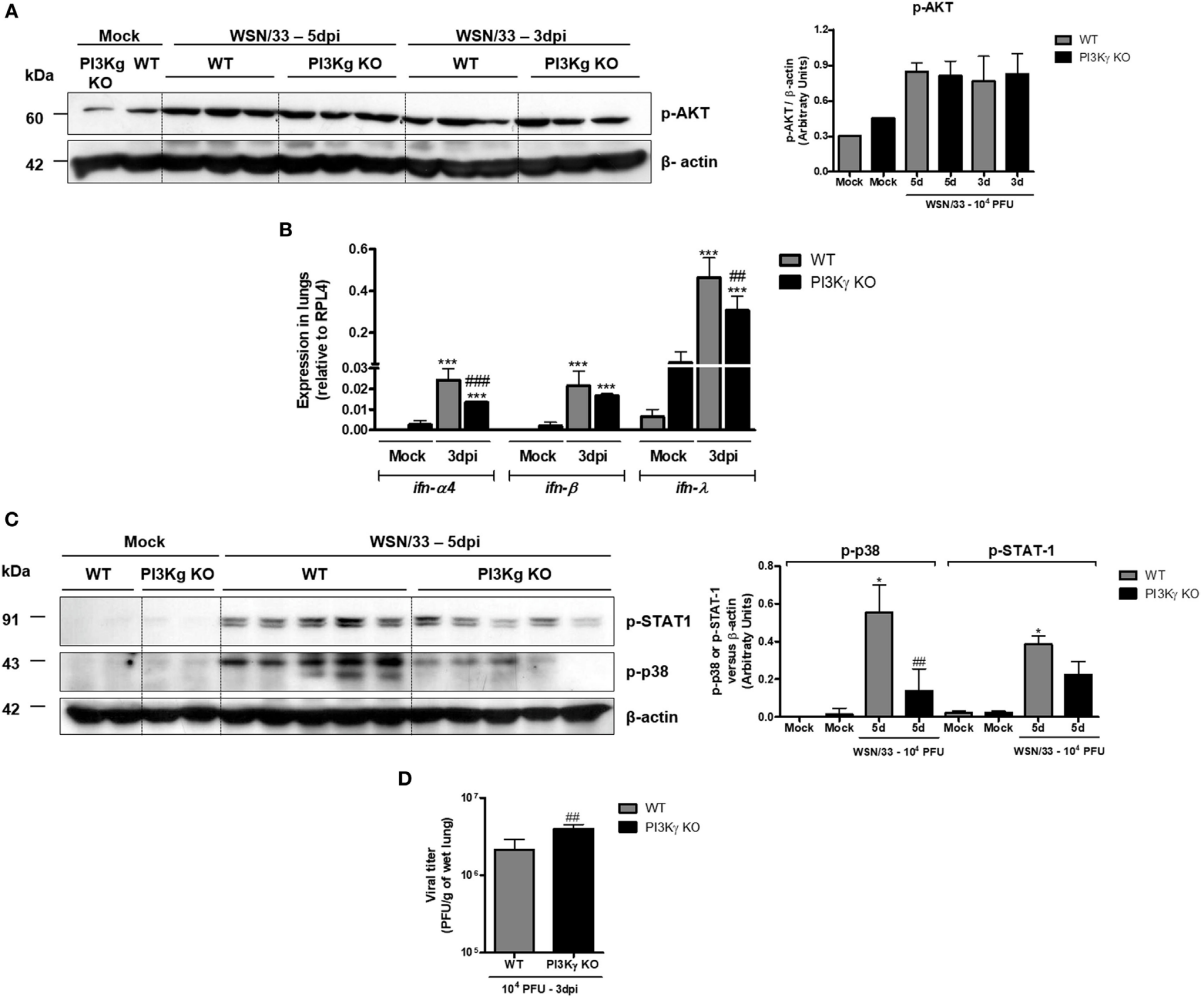
Reduced type-I and type-III IFN and p38 activation in phosphatidyl inositol 3 kinase-gamma (PI3Kγ) knockout (KO) mice infected with influenza A virus are related to higher viral loads in early time points. WT and PI3Kγ KO mice were instilled with phosphate-buffered saline or 10^4^ plaque-forming units (PFU) of influenza WSN and euthanized at day 3 or 5 after infection (representative of two experiments showing *n* = 4–5 mice per group). Phosphorylation of **(A)** AKT in lung extracts from WT and KO mice at 3 and 5 days after infection. **(B)** Lung mRNA levels of *ifn-*α*4, ifn-*β, and *ifn-*λ by real-time qPCR. **(C)** STAT-1 (upper) and p38 phosphorylation (middle) at day 5 after infection on the same membrane; β-actin was a loading control (lower); densitometry analysis was performed, comparing signals of analyzed protein versus loading control (right). **(D)** Viral loads in lungs were assessed by plaque assay in Madin–Darby Canine Kidney cells at day 3 after infection. Results are presented as mean ± SD. ***p < 0.001, when compared with each Mock group; ^##^*p* < 0.01 and ^###^*p* < 0.001, comparing PI3Kγ KO to WT-infected mice [**(B)** one-way ANOVA, Newman–Keuls; **(D)** *t*-test].

It has been demonstrated that influenza RNA stimulates PI3K activation and consequent AKT phosphorylation that are important to innate responses and cell survival (33). Accordingly, we observed strong AKT phosphorylation in lungs from both WT- and PI3Kγ KO-infected mice at day 5 after infection (Figure 3A), suggesting that the virus preferentially activates the other PI3K isoforms during infection, which then induces AKT activation. Despite of the increased neutrophilic infiltration (Figure 2), and similar AKT phosphorylation (Figure 3A), PI3Kγ KO IAV-infected mice displayed impaired innate antiviral response. Remarkably, although the expression of *ifn-*α*4, ifn-*β, and *ifn-*λ has been found increased in lungs of WT and PI3Kγ KO mice (Figure 3B), the expression of *ifn-*α*4* and *ifn-*λ (Figure 3B) was lower in PI3Kγ KO mice, when compared with WT-infected mice. Therefore, in the absence of PI3Kγ there are decreased induction of type-I and type-III interferons, which in turn might impair the antiviral response of PI3Kγ KO mice.

The activation of IFNAR, by IFN-α or IFN-β, and IFNLR1 by IFN-λ results in downstream signaling that involves JAK/STAT cascade to induce antiviral effects (34). STAT-1 was strongly activated following IAV infection at day 5 in WT mice but not in PI3Kγ KO mice (Figure 3C, upper blot). Moreover, we found that p38 phosphorylation, which, together with PI3K, is pivotal for STAT-1 activation (35), was enhanced at day 5 after IAV infection in WT mice, but not in PI3Kγ KO mice, with a difference between infected groups (Figure 3C, lower). Accordingly to reduced type-I and type-III IFN production and reduced p38 activation in PI3Kγ KO mice, the viral loads in lungs were higher in KO mice at the beginning of infection when compared with WT mice (Figure 3D). Therefore, PI3Kγ seems to be important for innate antiviral responses against IAV infection.

### PI3Kγ Activation on BMDM Leads to ISG15 Production During IAV Infection in a p38-Dependent Way

**Figure 4:**
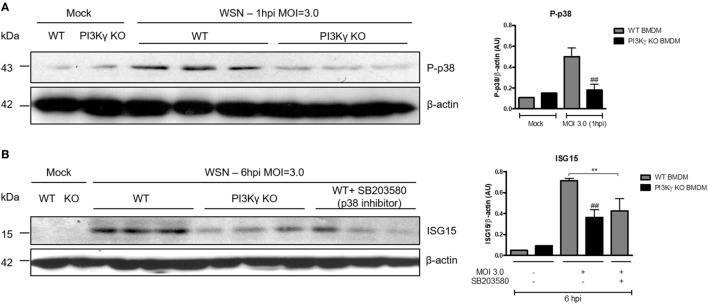
Phosphatidyl inositol 3 kinase-gamma (PI3Kγ) activation on bone marrow-derived macrophages (BMDM) leads to interferon-stimulated gene 15 (ISG15) production during influenza A virus infection in a p38-dependent way. BMDM from WT and PI3Kγ knockout (KO) were infected with influenza WSN [multiplicity of infection (MOI): 3.0]. **(A)** One hour after infection, BMBM protein extracts were analyzed for p38 phosphorylation; β-actin was a loading control. **(B)** WT BMDM were pretreated with the p38 inhibitor SB203580 or vehicle and KO BMDM were treated with vehicle then infected with WSN (MOI: 3.0). ISG15 protein expression was assessed at 6 h after infection; β-actin was a loading control. Results are presented as mean ± SD. **p < 0.01, when comparing WT-infected cells treated or not with SB203580; ^##^*p* < 0.01, comparing PI3Kγ KO to WT infected BMDM (one-way ANOVA, Newman–Keuls).

To elucidate the interplay between PI3Kγ and p38 as an antiviral mechanism, we performed *in vitro* infection of BMDM with WSN virus from WT and PI3Kγ KO mice. Similar to the *in vivo* infection (Figure 3C), IAV infection induced p38 phosphorylation in WT but not in PI3Kγ KO BMDM (Figure 4A). Therefore, we investigated the effect of p38 activation on the antiviral protein ISG15. Using the same BMDM *in vitro* system, WT-infected cells were treated with the p38 inhibitor SB203580 or treated with drug vehicle before and during WSN infection. In parallel PI3Kγ KO cells were infected and harvested 8 h after infection to evaluate the downstream antiviral molecule ISG15. Protein levels of ISG15 were induced during IAV infection in WT cells and were reduced in PI3Kγ KO cells and WT cells treated with the p38 inhibitor (Figure 4B). Thus, our results showed that p38 and PI3Kγ activation regulates the production of the antiviral protein ISG15 during IAV.

### Imbalanced Inflammatory and Antiviral Responses to IAV Infection in the Absence of PI3Kγ Are Not Cell-Type Dependent

**Figure 5:**
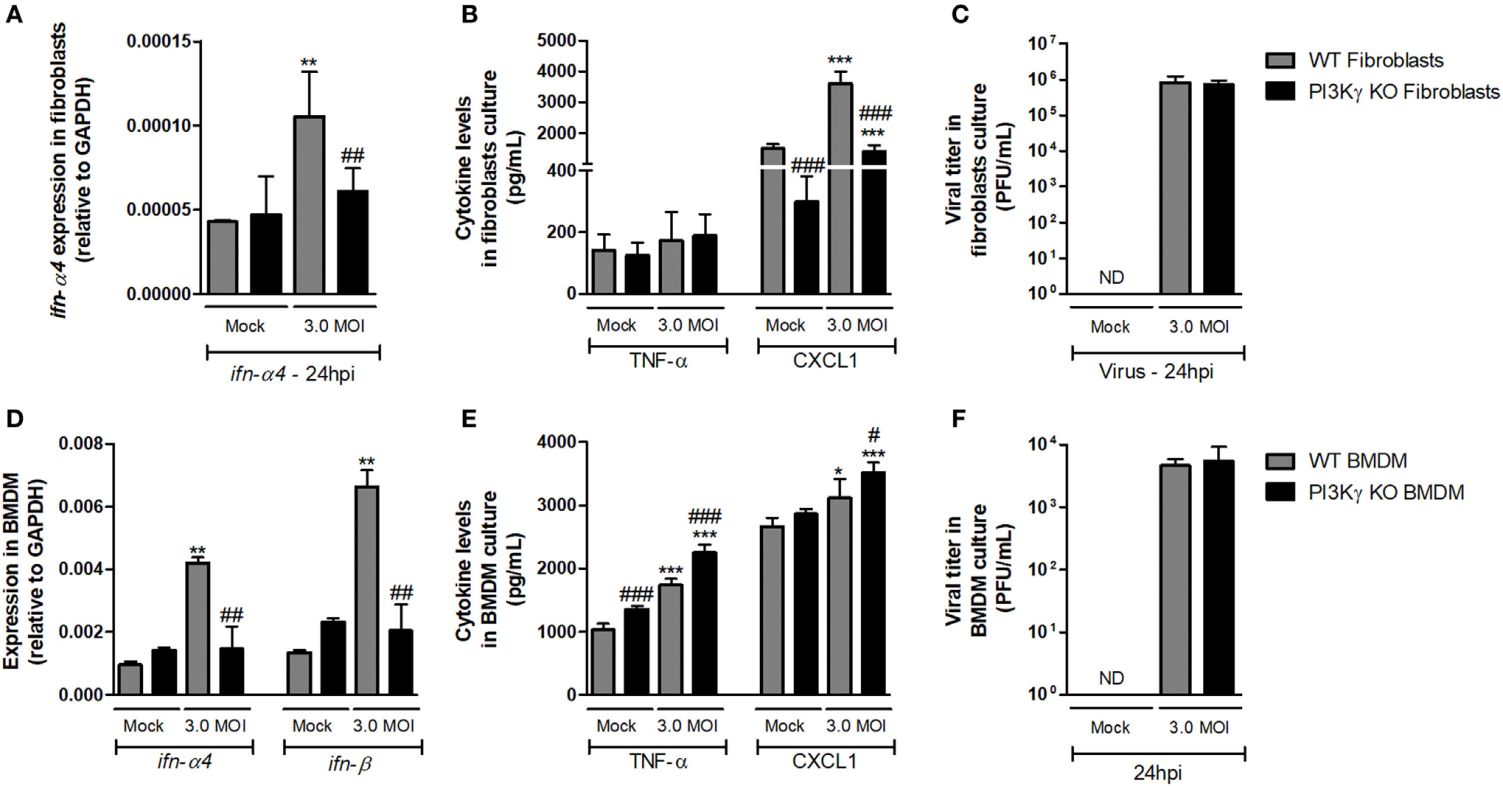
Altered antiviral responses in the absence of phosphatidyl inositol 3 kinase-gamma (PI3Kγ) in leukocytes and resident cells. Lung-derived fibroblasts from WT and PI3Kγ knockout (KO) infected with influenza WSN [multiplicity of infection (MOI): 3.0] were evaluated after 24 h. mRNA levels of **(A)** *ifn-*α*4*, assessed by real-time PCR. In cell supernatants, **(B)** TNF-α and CXCL1 levels by ELISA and **(C)** viral titer in by plaque assay in Madin–Darby Canine Kidney cells. Bone marrow-derived macrophages (BMDM) from WT and PI3Kγ KO were infected with influenza WSN (MOI: 3.0). 24 h after infection, **(D)** *ifn-*α*4* and *ifn-*β expression, **(E)** TNF-α and CXCL1 levels, and **(F)** viral titer were assessed as for fibroblasts. Results are presented as mean ± SD. *p < 0.05, **p < 0.01, and ***p < 0.001, when compared with each Mock group; ^#^*p* < 0.05, ^##^*p* < 0.01, and ^###^*p* < 0.001, comparing PI3Kγ KO to WT-infected cells (one-way ANOVA, Newman–Keuls).

With the aim of elucidating whether mechanisms involved in regulation of antiviral response by PI3Kγ were cell dependent, we performed *in vitro* infection of lung-derived fibroblasts and BMDM with WSN virus. WT fibroblast infection induced expression of *ifn-*α*4* (Figure 5A) 24 h after infection, but PI3Kγ KO fibroblasts did not. In addition, no induction in TNF-α was detected in WT and PI3Kγ KO fibroblasts, while CXCL1 was induced by the infection in KO cells, although in lower levels (Figure 5B). No differences in viral loads were detected in WT and PI3Kγ KO fibroblasts at 24 h after IAV infection (Figure 5C). Similarly to fibroblasts, IAV infection of BMDM induced *ifn-*α*4* and *ifn-*β expression in WT but not in PI3Kγ KO cells (Figure 5D). Contrastingly, BMDM produced high levels of TNF-α upon WSN infection. Moreover, this induction was higher in KO cells. Finally, CXCL1 was induced only in KO BMDM after IAV infection (Figure 5E). Interestingly, despite the reduced type-I IFN induction, similar viral levels were found in WT and PI3Kγ KO BMDM (Figure 5F). Altogether, these results show that the absence of PI3Kγ negatively affects type-I IFN response in either resident cells (fibroblasts) or leukocytes (BMDM), but the production of inflammatory mediators is differently regulated in either cell type. However, direct control of viral replication is not affected by PI3Kγ in a cell autonomous manner in the evaluated cell populations. Thus, increased viral replication in the lungs of KO-infected mice might be dependent on a combination of both cell autonomous and cell–cell communication defects that result in a distinct inflammatory milieu.

### Impaired Late Antiviral Response in the Absence of PI3Kγ

**Figure 6:**
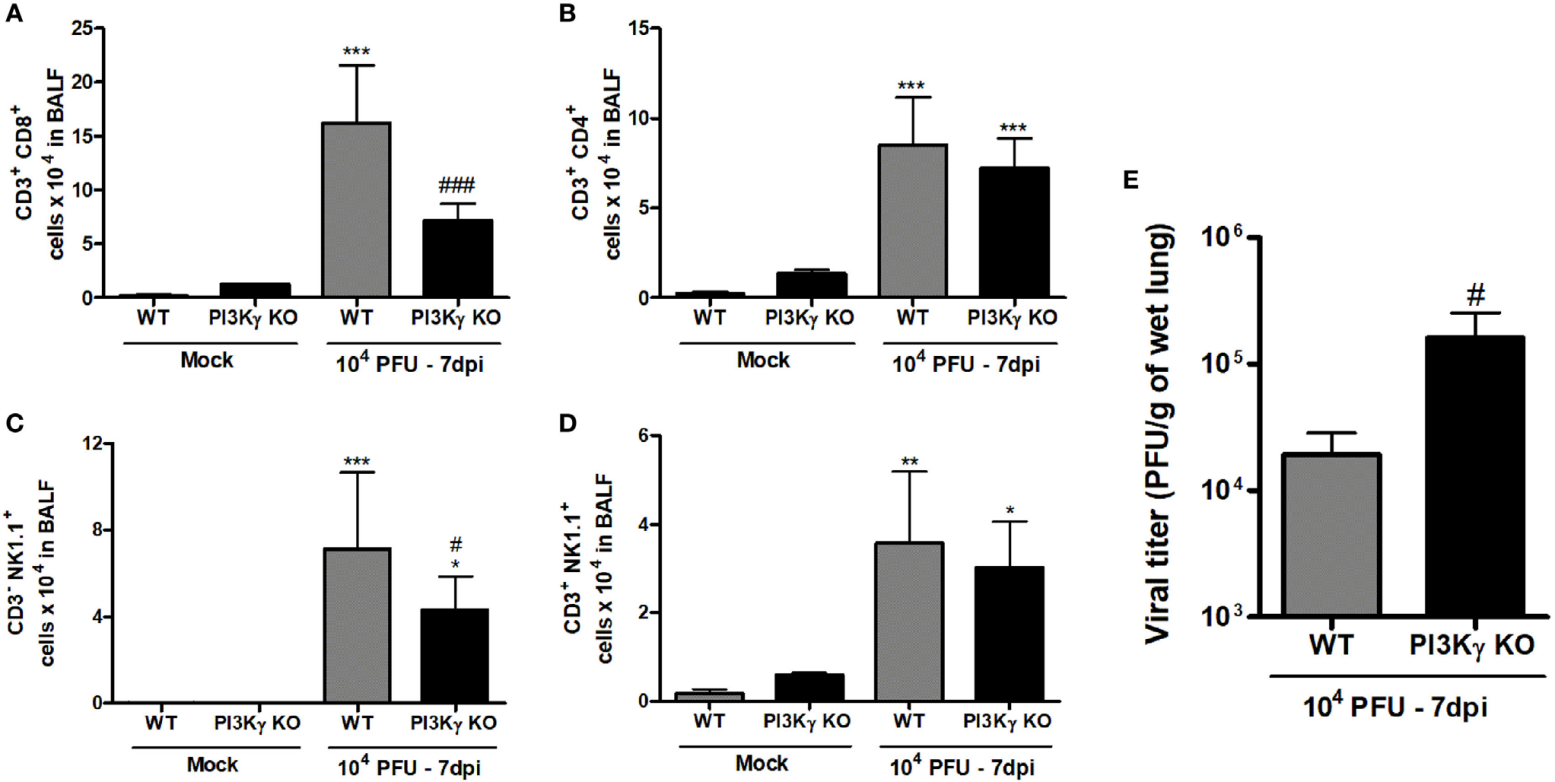
Late lymphocyte infiltration and viral control in lungs of WT and phosphatidyl inositol 3 kinase-gamma (PI3Kγ) knockout (KO) after influenza A virus infection. WT and PI3Kγ KO mice were inoculated with 10^4^ plaque-forming units (PFU) of influenza WSN or phosphate-buffered saline and killed after 7 days (representative of two experiments showing *n* = 4–7 mice per group). Number of **(A)** CD8^+^ T cells (CD3^+^CD8^+^), **(B)** CD4^+^ T cells (CD3^+^CD4^+^), **(C)** natural killer (NK) cells (CD3^−^NK1.1^+^), and **(D)** NKT cells (CD3^+^NK1.1^+^) were evaluated in bronchoalveolar lavage fluid (BALF) by flow cytometry. **(E)** Viral levels in lungs, assessed by plaque assay in Madin–Darby Canine Kidney cells. Results are presented as mean ± SD. *p < 0.05, **p < 0.01, and ***p < 0.001, when compared with Mock groups; ^#^*p* < 0.05 and ^###^*p* < 0.001, comparing KO to WT-infected mice [**(A–D)** one-way ANOVA, Newman–Keuls; **(E)** *t*-test].

During IAV infection, NK cells and T lymphocytes are important effector cells for the clearance of the virus (8). Therefore, we analyzed the populations of lymphocytes and NK cells in airways of WT and PI3Kγ KO mice in a late phase (7 days) of infection, when adaptive responses were already established. We found higher numbers of CD8^+^ T cells in WT mice than in PI3Kγ KO mice (Figure 6A), whereas the number of CD4^+^ T cells was similar in WT and PI3Kγ KO-infected mice (Figure 6B). In addition, the number of NK (Figure 6C) and NKT cells (Figure 6D) was greater after IAV infection in WT mice. In PI3Kγ KO mice, NK cells were found in lower numbers after infection than in WT-infected mice (Figure 6C) but NKT cells were at similar numbers to WT mice (Figure 6D). No differences in B cell population (CD19^+^B220^+^) were found between Mock and infected mice (data not shown). Finally, higher viral loads were found in lungs of PI3Kγ KO-infected mice at day 7 (Figure 6E), strongly suggesting that reduction in the recruitment of CD8^+^ T cells and NK cells, two important cell populations for viral clearance, might impair this process in lungs of PI3Kγ KO mice, as compared with WT mice. Taken together, the results show that PI3Kγ plays an important role in both innate and adaptive antiviral response against IAV.

### Altered Pro-Resolving Profile of Airway Leukocytes of PI3Kγ KO Mice

**Figure 7:**
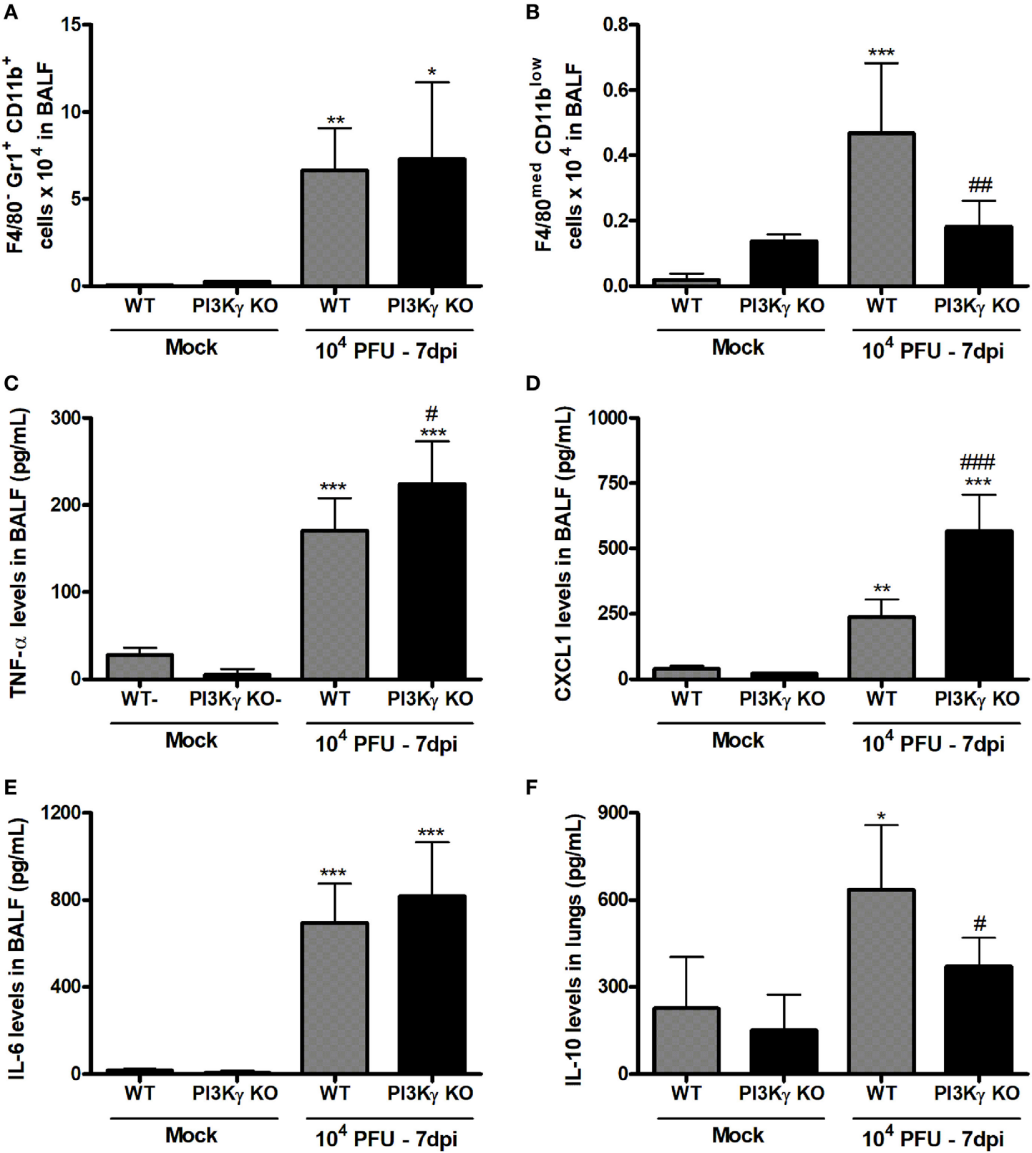
Unbalanced inflammatory pro-resolving responses in phosphatidyl inositol 3 kinase-gamma (PI3Kγ) knockout (KO) infected with influenza A virus. WT and PI3Kγ KO mice were instilled with phosphate-buffered saline or WSN virus and euthanized at 7 days after infection (representative of two experiments showing *n* = 4–7 mice per group). FACS analysis in airways leukocytes showed **(A)** numbers of neutrophils (F4/80^−^Gr1^+^CD11b^+^) and **(B)** resolving macrophages (F4/80^med^CD11b^low^). Levels of **(C)** TNF-α, **(D)** CXCL1, and **(E)** IL-6 in bronchoalveolar lavage fluid (BALF) and from **(F)** IL-10 in the lungs were assessed by ELISA. Results are presented as mean ± SD. *p < 0.05, **p < 0.01, and ***p < 0.001, when compared with each Mock group; ^#^*p* < 0.05, ^##^*p* < 0.01, and ^###^*p* < 0.001, comparing PI3Kγ KO- to WT-infected mice (one-way ANOVA, Newman–Keuls).

Besides antiviral adaptive immunity, we also evaluated the involvement of PI3Kγ on late stages of the inflammatory responses triggered by IAV infection. After 7 days of infection, when PI3Kγ KO mice usually start to die, the number of neutrophils (F4/80^−^Gr1^+^CD11b^+^) was equally increased in the airways of WT and PI3Kγ KO mice (Figure 7A). On the other hand, the recruitment of macrophages (F4/80^+^ cells) into airways induced by IAV infection was reduced in PI3Kγ KO mice compared with WT (data not shown). We further analyzed the profile of this macrophage population based on three macrophages populations: M1-like (F4/80^low^Gr1^+^CD11b^med^), M2-like (F4/80^high^Gr1^−^CD11b^high^), and Mres (F4/80^med^CD11b^low^) (36–39). Thus, among the three populations, Mres (Figure 7B), but not M1 or M2 (data not shown) was found increased in BALF of WT mice but not in PI3Kγ deficient mice at day 7 after IAV infection. It is noteworthy that Mres are involved in resolution of inflammation and tissue repair (40). Therefore, their lower numbers in airways of PI3Kγ KO mice may hence contribute to a more intense and harmful lung inflammation.

Evaluation of pro-inflammatory and anti-inflammatory cytokines locally produced and recovered from BALF 7 days after infection showed that the pro-inflammatory cytokine TNF-α and the chemokine CXCL1, important for neutrophil recruitment, were increased in WT and PI3Kγ KO mice, albeit in higher levels in PI3Kγ KO mice (Figures 7C,D). In contrast, IL-6 levels were equally increased at day 7 after infection in WT and PI3Kγ KO mice (Figure 7E). Thus, in the lack of PI3Kγ signaling in KO mice, the response to CXCL1 gradient in the airways would lead to similar recruitment of neutrophils into the airways (Figure 7A) in WT and KO mice, as previously shown (41). On the other side of inflammatory balance, the cytokine IL-10, known to be anti-inflammatory, was increased at this later time after IAV infection in lungs of WT, but not in PI3Kγ KO mice (Figure 7F). The lower IL-10 levels could be a consequence of the reduction in the number of pro-resolving macrophages and the poor prognosis of PI3Kγ KO mice (Figure 7B).

### Increased Neutrophil Accumulation, Activation, and Lung Damage in PI3Kγ KO Mice

**Figure 8:**
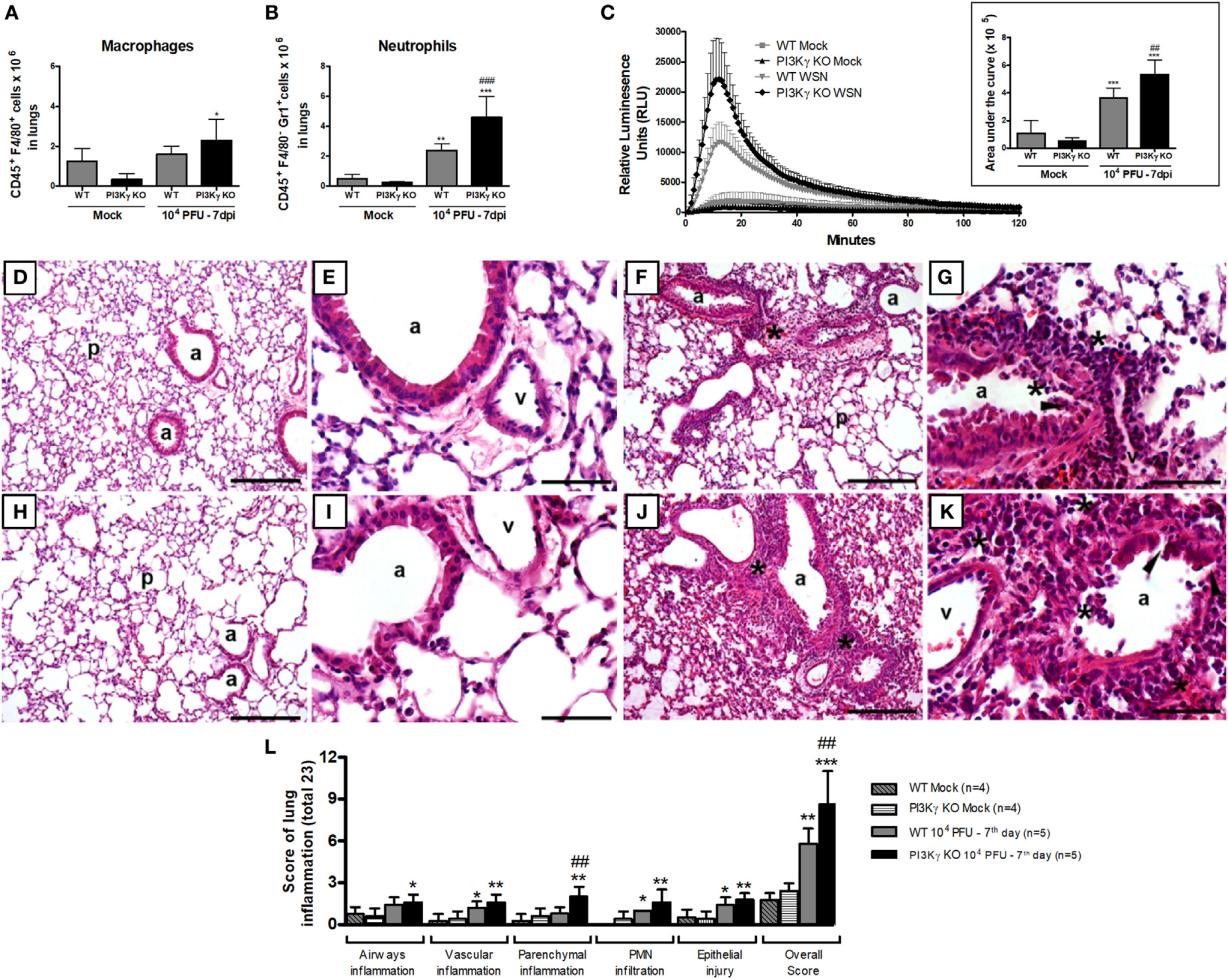
Neutrophil recruitment and activation and lung damage during influenza A virus infection are affected by phosphatidyl inositol 3 kinase-gamma (PI3Kγ). WT and PI3Kγ knockout (KO) mice infected with 10^4^ plaque-forming units (PFU) of WSN or receiving phosphate-buffered saline (Mock) were killed at day 7 after infection (*n* = 4–7 mice per group). **(A)** Macrophage (CD45^+^F4/80^+^) and **(B)** neutrophils (CD45^+^F4/80^−^GR1^+^) numbers in lungs assessed by flow cytometry. **(C)** Reactive oxygen species assessed in lung leukocytes in presence of luminol with or without zymosan for 120 min. Relative luminescence units mean the value with zymosan minus the respective value without zymosan. Areas under the curve were compared using mean ± SEM and one-way ANOVA, Newman–Keuls test **(D–K)**. Representative slides of hematoxylin and eosin stained lung sections from WT **(D,E)** and PI3Kγ KO mock **(H,I)**, WT **(F,G)**, and PI3Kγ KO-infected mice **(J,K)** are shown in 100× [**(D,H,F,J)**, bars = 200 μm] and 400× [**(E,I,G,K)**, bars = 50 μm] magnification. “p” Indicates lung parenchyma, “v” vessels, and “a” airways; asterisks foci of inflammatory infiltrates; and arrowheads areas of epithelial injury. **(L)** Histopathological score (total 23 points) assessed airway, vascular, parenchymal inflammation, neutrophilic infiltration, and epithelial injury. Data are presented as mean ± SD. *p < 0.05, **p < 0.01, and ***p < 0.001, compared with each Mock group; ^##^*p* < 0.01 and ^###^*p* < 0.001, compared with WT-infected mice (one-way ANOVA, Newman–Keuls).

To better understand the reasons by which PI3Kγ KO mice were more susceptible to influenza-induced lethality, leukocytes were extracted from the lungs of Mock and IAV-infected WT and PI3Kγ KO mice 7 days after infection to analyze granulocyte populations and their activation. FACS analysis of CD45^+^ cells recovered from lungs showed that macrophage (F4/80^+^) recruitment to the lungs was not increased during IAV infection after 7 days in WT but it was increased in PI3Kγ KO mice (Figure 8A). Likewise, a remarkable increase of neutrophil numbers (Gr1^+^F4/80^−^) was observed at day 7 after infection in the lungs of PI3Kγ KO mice compared with WT (Figure 8B).

Activation of neutrophils and macrophages results in the release of reactive oxygen species (ROS), an important mechanism to kill intracellular pathogens, such as bacteria and protozoa, but that may also cause tissue damage under inflammatory conditions (42). To assess ROS production, we performed a chemiluminescence assay in lung leukocytes using the TLR2/6 ligand zymosan as a stimulus. The production of ROS was increased in leukocytes isolated from lungs of infected WT mice and the peak of detection was five times higher, when compared with the Mock WT mice (Figure 8C). In leukocytes extracted from PI3Kγ KO-infected mice, we detected an ROS peak 20 times higher compared with Mock KO mice. Comparing ROS production at day 7 after IAV infection, PI3Kγ KO cells produced higher amounts of ROS than WT cells (Figure 8C). The increased ROS production was most likely released by neutrophils rather than macrophages, because the number (Figure 8A) and percentage of Gr1^+^F4/80^−^ cells among CD45^+^ cells was increased in PI3Kγ KO-infected mice (WT 33 ± 5%; KO 46 ± 9%) and was also higher than F4/80^+^ cells.

To assess the impairment of pulmonary structures 7 days after IAV infection in WT and PI3Kγ KO mice, we evaluated H&E stained lung tissue slides for airway, vascular and parenchyma inflammation, PMN infiltration and epithelial injury. WT (Figures 8D,E) and PI3Kg KO (Figures 8H,I) mock mice did not present significant inflammatory changes in lung slides. IAV infection resulted in epithelial injury and infiltration of leukocytes, mainly neutrophils, especially close to the vessels in WT mice (Figures 8F,G). These inflammatory changes were more pronounced in PI3Kγ KO-infected mice, which also presented inflammation in the airways and more intense parenchymal inflammation, resulting in a significantly higher overall histopathological score than WT-infected mice (Figures 8J,K). Although vascular inflammation and epithelial damage were similar in both infected groups (Figure 8L), the overall score was higher in PI3Kγ KO mice. Taking together, an early impairment of antiviral response, a subsequent decreased infiltration of CD8^+^ lymphocytes, NK cells, and resolving macrophages, concurrent with enhanced neutrophilic infiltration culminates with higher viral loads and increased ROS production that contributes to more pronounced histopathological injuries found in lungs of PI3Kγ KO mice, which might explain the increased lethality rates found in the KO group compared with WT mice upon IAV infection.

### Genetic Polymorphisms in *PIK3CG* Are Associated With Disease Outcome in Influenza-Infected Patients

The evaluation of three *PIK3CG* SNPs (rs1129293, rs17847825, and rs2230460) was carried out in influenza A(H1N1)pdm09-infected patients, who were further stratified according to their clinical manifestations or outcomes as ILI, SARI, and deceased. A descriptive analysis of the available epidemiological data of the recruited patients is shown in Table 2. There were significant differences of mean age, gender, influenza vaccination, oseltamivir treatment and comorbidities (pneumopathy, cardiopathy, immunodepression, hypertension, hepatopathy, neurologic disorders, nephropathy, diabetes, cancer, obesity, and smoking) among groups. It is important to highlight that the information regarding oseltamivir treatment, influenza vaccination, and the presence of comorbidities was not always available in the patients’ records.

**Table 2:** Descriptive data of clinical characteristics of H1N1-infected patients enrolled in the study of genetic association of PIK3CG polymorphisms.

Variable^a^	ILI, *N* = 121	SARI, *N* = 137	Deceased, *N* = 92	*p*-Value
Age, mean ± SD, years	29.7 ± 19.4	40.7 ± 23.5	41.3 ± 20.1	Kruskal–Wallis <0.001
Sex, male (%)	71 (58.7)	60 (43.8)	52 (56.5)	Chisq. 0.037
Major comorbidity (%)	12 (34.3)	58 (62.4)	16 (59.3)	Chisq. 0.016
Onset-to-presentation, mean ± SD, days	3 ± 3.3	4 ± 3.6	6 ± 4.2	Kruskal–Wallis <0.001
Hospitalization (%)	0 (0)	131 (97.8)	52 (96.3)	Fisher 0.028
Oseltamivir treatment (%)	20 (45.5)	81 (80.2)	27 (51.9)	Chisq. <0.001
Flu vaccine (%)	9 (12.5)	29 (27.6)	3 (10.3)	Fisher 0.03

*ILI, influenza-like illness; SARI, severe acute respiratory infection*.

*^a^Clinical variable of H1N1-infected patients*.

Genotyping results indicated a call rate efficiency higher than 95% for the three tested SNPs. Using a regression model including sex and age as categorical variables (Table 3), we found a significant association results for rs17847825 AA genotype [odds ratio (OR) = 0.1, *p* = 0.03] under codominant and also recessive model (OR = 0.1, *p* = 0.007) when comparing ILI versus the combination of SARI and Deceased. The rs2230460 TT genotype also had a borderline association in the codominant (OR = 0.1, *p* = 0.07) and recessive genetic models (OR = 0.1, *p* = 0.02). No significant associations were detected for the rs1129293 C>T SNP. These results suggest that both rs17847825 and rs2230460 polymorphisms in PI3Kγ could be playing protective roles in the context of infection and decreasing the likelihood of progression toward the more severe manifestations of the disease.

**Table 3:** Analysis of SNPs located in *PIK3CG*.

	ILI, *N* = 121	SARI,*N* = 137	Deceased,*N* = 92	ILI versus SARI		ILI versus deceased		ILI versus SARI + deceased		SARI versus deceased	
				OR^a^ [95% CI]	*p*-Value	OR^a^ [95% CI]	*p*-Value	OR^a^ [95% CI]	*p*-Value	OR^a^ [95% CI]	*p*-Value
**rs1129293 C>T**											
*Codominant*											
CC	64 (0.53)	71 (0.52)	39 (0.42)	Reference							
CT	48 (0.40)	55 (0.40)	47 (0.51)	1.0 [0.6–1.7]	0.74	1.7 [0.9–3.0]	0.22	1.2 [0.8–1.9]	0.64	1.6 [0.9–2.8]	0.22
TT	8 (0.07)	11 (0.08)	6 (0.07)	1.5 [0.5–4.1]		1.2 [0.4–3.8]		1.4 [0.5–3.5]		1.0 [0.3–2.8]	
*Dominant*											
CT–TT	56 (0.47)	66 (0.48)	53 (0.58)	1.1 [0.6–1.8]	0.81	1.6 [0.9–2.8]	0.10	1.2 [0.8–1.9]	0.37	1.5 [0.9–2.5]	0.15
*Recessive*											
CC–CT	112 (0.93)	126 (0.92)	86 (0.94)	Reference							
TT	8 (0.07)	11 (0.08)	6 (0.06)	1.5 [0.5–4.0]	0.45	0.9 [0.3–2.8]	0.9	1.3 [0.5–3.1]	0.61	0.8 [0.3–2.2]	0.60

**rs17847825 A>C**											
*Codominant*											
CC	97 (0.80)	117 (0.85)	75 (0.82)	Reference							
CA	18 (0.15)	19 (0.14)	17 (0.19)	0.8 [0.4–1.7]	0.16	1.12 [0.5–2.4]	0.05	0.9 [0.5–1.8]	**0.03**	1.4 [0.7–2.8]	0.48
AA	6 (0.05)	1 (0.01)	0 (0.0)	0.1 [0.1–1.3]		NA		**0.1 [0.1–0.8]**		NA	
*Dominant*											
CA–AA	24 (0.20)	20 (0.15)	17 (0.18)	0.7 [0.3–1.3]	0.22	0.9 [0.4–1.8]	0.69	0.7 [0.4–1.3]	0.31	1.3 [0.6–2.7]	0.46
*Recessive*											
CC–AC	115 (0.95)	136 (0.99)	92 (1.0)	Reference							
AA	6 (0.05)	1 (0.01)	0 (0.0)	**0.2 [0.1–1.4]**	**0.05**	NA		**0.1 [0.1–0.8]**	**0.007**	NA	

**rs2230460 C>T**											
*Codominant*											
CC	98 (0.82)	117 (0.85)	75 (0.82)	Reference							
CT	17 (0.14)	19 (0.14)	17 (0.18)	0.9 [0.4–1.8]	0.21	1.2 [0.6–2.6]	0.09	1.0 [0.5–1.9]	**0.07**	1.4 [0.7–2.8]	0.48
TT	5 (0.04)	1 (0.01)	0 (0.0)	0.2 [0.1–1.7]		NA		**0.1 [0.1–1.0]**		NA	
*Dominant*											
CT–TT	22 (0.18)	20 (0.15)	17 (0.18)	0.7 [0.4–1.5]	0.38	1.0 [0.5–2.0]	0.94	0.8 [0.4–1.5]	0.52	1.3 [0.6–2.7]	0.46
*Recessive*											
CC–CT	115 (0.96)	136 (0.99)	92 (1.00)	Reference							
TT	5 (0.04)	1 (0.01)	0 (0.0)	0.2 [0.1–1.7]	0.09	NA		**0.1 [0.1–1.0]**	**0.02**	NA	

*^a^Measure of association between clinical outcomes*.

*Significant associations are highlighted in bold*.

*Logistic regression results comparing between different clinical forms of influenza patients*.

*OR values shown are corrected for sex and age as non-genetic covariates*.

*Results are shown as *n* (frequency)*.

*CI, confidence interval; ILI, influenza-like illness; SARI, severe acute respiratory infection; OR, odds ratio; SNP; single-nucleotide polymorphism*.

*Total genotype counts can vary due to different genotypic call rates*.

## Discussion

Although PI3Kγ has been associated with exacerbated inflammatory responses in several models of inflammation, we demonstrated here that during IAV infection PI3Kγ plays a regulatory role. Our major findings were that the absence of PI3Kγ during IAV infection led to increased lethality and early neutrophil recruitment, in parallel to reduced early innate cell-independent p38-dependent antiviral response. Later, NK and CD8^+^ T lymphocytes also presented impaired recruitment leading to higher viral loads. Besides, increased production of pro-inflammatory mediators and reduction of resolving macrophages and IL-10 contributed to increased ROS production and lung damage that culminated with death of PI3Kγ KO mice. We also demonstrated the importance of PI3Kγ during influenza infection in a genetic association study showing that the SNPs rs17847825 and rs2230460 are protection factors of influenza clinical progression toward severe disease manifestations. Therefore, this is the first study that finds a genetic association of PI3Kγ variants and influenza severity. Furthermore, we showed for the first time the importance of PI3Kγ for the early antiviral response against IAV *via* p38 activation and for creating an anti-inflammatory/pro-resolving environment that protects from influenza-induced lung pathology.

Many studies about the role of PI3Kγ in inflammatory-driven diseases, have shown the potential of this enzyme as a promising therapeutic target (18). PI3Kγ KO mice were found to be more resistant to many types of experimental inflammatory diseases including pulmonary fibrosis (28), graft-versus-host disease (43), LPS-induced acute lung injury (ALI) (44), among others. On the other hand, absence or blockade of PI3Kγ worsened outcome in bacterial disease. For instance, after *Staphylococcus aureus* (45) or *Streptococcus pneumoniae* (46) infection in PI3Kγ KO mice there were less leukocyte recruitment and higher bacterial burden. The role of PI3Kγ in the context of viral infections has been studied in Kaposi’s sarcoma-associated herpes virus-induced tumors, where PI3Kγ is required for the viral oncogene signaling (47). During influenza A infection, the acute inflammatory response driven by the virus albeit detrimental to the host, contributes to trigger the adaptive immune response, which controls the infection. PI3Kγ has been shown to be important for the priming of CD8^+^ T cells by resident dendritic cell during influenza A infection and this regulation is important to virus control and resistance against infection induced lethality (24). In agreement with this study, we found an important role of PI3Kγ in regulating recruitment of CD8^+^ T to the lungs and to the control of the virus. We also showed that PI3Kγ is also important to regulate multiple mechanisms of innate immune responses, establishment and resolution of inflammation upon influenza infection.

The importance of PI3Kγ on early type-I and type-III IFN expression during influenza infection was associated with increased viral titers in PI3Kγ KO mice. BMDM and fibroblasts, that also express PI3Kγ (48), infected with the same influenza A strain also presented reduced type-I IFN expression in the absence of PI3Kγ, regardless of similar viral titers in the evaluated time points. It has already been described that when IAV enters the host cell, class IA PI3K is activated and stimulates IFN-β production (21) and that this activation is mediated by the viral RNA itself, not by the binding of the NS1 protein to PI3K (33). The study from Hrincius et al. (33) used a pan-inhibitor of PI3K (LY294002) to demonstrate that vRNA activates PI3K signaling causing IFN-β production. Therefore, a specific role of PI3Kγ could not be properly evaluated in that system. However, most of the studies regarding PI3K activation by IAV so far are *in vitro* (21–23, 33, 49, 50).

Absence of type-I IFN signaling during IAV infection increased lethality rates, viral loads, inflammation and lung damage in mice (51). IAV-infected type-I IFN receptor KO mice produced large amounts of CXCL1/KC and neutrophilia in addition to lung damage/lethality during IAV infection (51). This study is in accordance to our findings that in the absence of PI3Kγ with an early reduction of IAV-induced type-I and type-III IFN, there is increased production of pro-inflammatory cytokines and accumulation of activated neutrophils in the lungs that might contribute to lung damage and enhanced lethality.

It has been shown that PI3Ks and p38 MAPK may be necessary for the activation of STAT-1 and further ISG induction (35). We showed here that PI3Kγ was important for type-I and type-III IFN production, but *via* p38 activation, controlling ISG15 protein levels leading to an impaired antiviral activity responsible for innate viral clearance. Indeed, the use of p38 inhibitors during highly pathogenic IAV H5N1 infections lead to reduced type-I and type-III IFN, ISGs, and inflammatory cytokines expression (52, 53) that protects mice against lethality (53).

Phosphatidyl inositol 3 kinase/AKT activation is described to be important for signaling after binding of IFNs to their receptors and generation of antiviral activity (54). On the other hand, PI3Kα-β/AKT is also used by the IAV to promote cell survival and viral replication (22). A study demonstrated that, in cells from chronic obstructive pulmonary disease patients, PI3Kα levels are increased after influenza infection and its specific inhibition reduces AKT activation and viral infection by enhancing antiviral response (55). We showed that p-AKT levels were similar in WT and PI3Kγ KO mice after infection. Thus, a competition in the use of AKT by IFNAR activation and the virus itself might take part in the context of IAV infection (54). Therefore, we can infer that in the absence of PI3Kγ during influenza A infection, the production of type-I and type-III IFN is reduced and then, by hyperactivating PI3Kα, the virus uses the great availability of AKT for replication and on the other hand the reduced antiviral response will favor increased viral loads.

Besides this new role of PI3Kγ on innate antiviral immunity that we demonstrated in the present work, it is well known that PI3Kγ is involved in the recruitment and survival of leukocytes (45, 56). We found important differences in recruitment and activation of diverse leukocytes populations to distinct lung compartments and in leukocyte survival in PI3Kγ KO mice infected with IAV. There was an early increase in neutrophil transmigration to the airways and a late enhanced accumulation of these cells that are also highly activated in the lung parenchyma; on the other hand, the recruitment of macrophages, especially macrophages with resolving phenotype was reduced in the latest evaluated time point. PI3Kγ or PI3Kδ are important for neutrophil recruitment and are differently regulated upon distinct stimuli (41), thus IAV might induce neutrophil migration mainly *via* PI3Kδ but not PI3Kγ. Alternatively, one of the enzymes can account for the activity of the other, as effective reduction of recruitment is only achieved when both enzymes are inhibited or absent (41).

We also demonstrated that, in the absence of PI3Kγ, lymphocyte infiltration was reduced from day 5 after infection, with greatly reduced number of CD8^+^ T cells and NK cells at day 7. The infection with IAV leads to an early NK cell recruitment and late increase in the number of specific T cells against infected cells (57, 58). The reduction on priming of specific CD8^+^ T cells by dendritic cells on PI3Kγ KD/KD mice during IAV infection has been demonstrated (24). Besides DC mediated CD8^+^ T cell priming, other mechanisms might be involved in the reduced number CD8^+^ T cells in lungs of PI3Kγ-infected mice. One additional mechanism might be the multiple chemotactic signals generated in the lungs and airways cells after IAV infection that guide lymphocyte movement from draining lymph nodes to the site of infection (59). It has been demonstrated that during IAV infection, the migration of neutrophils into the airways leaves behind a trail containing the chemokine CXCL12 that guides the recruitment of CD8^+^ T cells toward the site of infection (60). We showed that neutrophil recruitment to the airways is similar or even increased in PI3Kγ absence, which would function as an important source of chemotactic stimuli for lymphocyte migration. For all these chemotactic signals, PI3Kγ might be a relevant downstream signaling module (61). Furthermore, PI3Kγ has already been demonstrated to promote lymphocyte migration toward chemokine stimuli, proliferation and differentiation (16, 61, 62). Thus, without proper antiviral NK and T cell responses, influenza virus could proliferate more in the absence of PI3Kγ and contribute to lethal infection.

Besides of leukocyte recruitment PI3Kγ might also be important to control leukocyte survival and resolution of inflammation in the context of IAV-induced inflammation as seen by the reduced number of resolving macrophages and lower IL-10 levels on KO mice. Resolving macrophages are generated from M2 macrophages after intensive efferocytosis and they are important sources of TGF-β and are more able to emigrate to lymphoid organs (36). Keeping with that, this unbalanced inflammatory response in PI3Kγ KO mice during IAV infection with the predominance of pro-inflammatory signals—cytokines, neutrophil infiltration and activation—and reduced anti-inflammatory/pro-resolving signals—Mres and IL-10—may account to the increased lung injury in PI3Kγ KO mice.

The enhanced lethality of PI3Kγ KO mice is a combination of a fail on viral control and an enhanced inflammation and lung damage, mainly caused by increased neutrophilic infiltration and ROS production in lungs. Production of ROS by leukocytes during influenza infection is known to contribute to the ALI in the most severe cases (63). Reducing ROS production using mutant mice for different components of the NADPH oxidase complex like ncf1 (63) or Nox2 oxidase (64) results in better outcome following infection with different strains of IAV. Here we describe that leukocytes from PI3Kγ KO mice infected with WSN produced more ROS when stimulated with zymosan. Nevertheless, it has been already described that PI3Kγ is important for ROS generation under GPCR stimulation, including that by the bacterial component *N*-formylmethionyl-leucyl-phenylalanine (fMLP) (65). However, zymosan does not trigger GPCR and GPCR-triggered respiratory burst is not totally dependent on PI3Kγ (16). A deeper evaluation of the generation of ROS by neutrophils induced by fMLP demonstrates that ROS production is enhanced by the presence of cytokines such as TNF-α in later time points (66) and a more complex mechanism involving the class IA PI3Ks, especially PI3Kβ (67) or by direct activation of NADPH oxidase by GPCR-induced Src family kinases (68). Other stimuli that do not activate GPCRs can also activate cells to produce ROS, including TLR agonists, such as zymosan (69), the cytokines TNF-α and GMCSF (70) or the virus itself (71). Thus, the increased inflammatory environment found in PI3Kγ KO mice after IAV infection, including more TNF-α and more virus loads might contribute to ROS generation and the increased lung damage found in these mice with consequent higher death rates.

Severe human influenza cases are associated with delayed peripheral immune activation and prolonged inflammation, compared with mild influenza cases (72). We observed this prolonged inflammation in PI3Kγ KO mice leading to tissue injury. Therefore, we aimed to correlate the important functional role of PI3Kγ during influenza A infection demonstrated in the murine model to a genetic association study. In this study, we used clinical samples from A(H1N1)pdm09-infected patients presenting with mild (ILI) or severe (SARI) manifestations and also from deceased patients to genotype three *PIK3CG* SNPs. The *PIK3CG* gene presents 85 SNPs on exons according to the 1000 Genomes project (32). Among them, 48 lead to amino acid changes or missense substitutions. However, AF of most of these SNPs is extremely low (<1%), requiring large sample size to detect a true genetic effect. We assessed *PIK3CG* SNPs MAF on a big database of SNPs from three distinct Brazilian cohorts (EPIGEN-Brazil Project) (31) and then selected three SNPs—rs1129293, rs17847825, and rs2230460—that presented MAF of 24, 8.6, and 8.8% according to EPIGEN-Brazil database. From the three SNPs, only rs17847825 leads to a predicted missense substitution (S/Y) with possible deleterious effect (Table 1). We found a protective association against severity progression for rs17847825 and rs2230460 SNPs but not rs1129293. The rs17847825 locates on the C2 domain of the PI3Kγ protein, which has a role on cellular membrane binding (73). However, it is not possible to say how this change from serine to tyrosine would impact positively or negatively on protein function. Interestingly, although rs2230460 represents a synonymous SNP, it does not exclude its role as a possible tag polymorphism that could be signaling that the protection association found here is led by another causal SNP that is in linkage disequilibrium with rs2230460.

Other SNPs on *PIK3CG* or close to the gene have been studied in genetic association studies. rs17398575, rs17477177, and rs342286 are located near *PIK3CG* and were associated with cardiovascular diseases risk (74–76). The *PIK3CG* SNP rs342286 was associated with epinephrine-induced aggregation (77) and the SNPs rs4288294 and rs116697954 associate with higher HDL2 cholesterol plasma levels and rs1129293, also evaluated in our study, was associated with lower HDL levels (78). Genetic association studies have been extensively used to search host factors that predispose to severe outcomes during influenza infection [reviewed in Ref. (79, 80)]. Here we showed for the first time a genetic association of *PIK3CG* SNPs on influenza protection. Further studies in distinct populations might be necessary to replicate our findings and to better elucidate the role of these mutations on protein function.

This work suggests that targeting PI3Kγ may not be useful to treat respiratory diseases caused by IAV, due to an increase of disease severity and decreased ability to control infection. Mechanistically, PI3Kγ is required for early type-I IFN production and ISG15 production *via* p38 activation, and for the migration of immune cells (NK and CD8^+^ T cells) necessary for viral replication control. On the other hand, PI3Kγ controls neutrophilic infiltrations and the resolution of inflammation. To sum up, PI3Kγ orchestrates, by distinct mechanisms, the antiviral immunity and inflammatory magnitude in response to IAV and might be an important marker of disease severity.

## Ethics Statement

All the procedures were performed according to the Guide for the Care and Use of Laboratory Animals of the Brazilian National Council of Animal Experimentation and the Federal Law 11.794 (October 8, 2008) and were previously approved by the local ethics committee (Comissão de Ética no Uso de Animais from Universidade Federal de Minas Gerais, protocol number 203/08). The use of clinical samples from the Respiratory Viruses and Measles Laboratory—the National Reference Laboratory for Influenza for the Brazilian Ministry of Health and a National Influenza Center from World Health Organization (WHO) in the network for influenza surveillance—was carried out in accordance with the recommendations of Resolution 466 (December 12, 2012) from the Brazilian National Health Board. The protocol was reviewed by the local Ethics in Research Committee at Instituto Oswaldo Cruz (Comitê de Ética em Pesquisa Approval number CAAE 68118417.6.0000.5248). The informed consent from the subjects was waived because the samples were originally for influenza diagnostics of the National Influenza Epidemiological Surveillance Program, as part of a global and federal public health policy for influenza control and prevention. All data corresponding to patients were analyzed to ensure anonymity.

## Author Contributions

CG, LT, AD, FK, LA-A, AMVM, LS, and RR participated on study’s conception, design, and experimental and analytical analysis; CQ-J, ARM, IG, BL, and AG participated on experimental and analytical analysis; FS, JM, MM, MS, and MT participated on study’s conception, design, and analytical analysis.

## Conflict of Interest Statement

The authors declare that the research was conducted in the absence of any commercial or financial relationships that could be construed as a potential conflict of interest.

## Supplementary Material

Figure S1 Gating strategies for FACS analysis of lymphocytes (1), natural killer (NK) and B cells (2), macrophages and neutrophils in bronchoalveolar lavage fluid (3), and macrophages and neutrophils in lungs (4).

## References

[1] Iuliano AD Roguski KM Chang HH Muscatello DJ Palekar R Tempia S Estimates of global seasonal influenza-associated respiratory mortality: a modelling study. Lancet (2018) 391(10127):1285–300. 10.1016/S0140-6736(17)33293-2 29248255 PMC5935243

[2] Bouvier NM Palese P. The biology of influenza viruses. Vaccine (2008) 26(Suppl 4):D49–53. 10.1016/j.vaccine.2008.07.039 19230160 PMC3074182

[3] Tumpey TM Garcia-Sastre A Taubenberger JK Palese P Swayne DE Pantin-Jackwood MJ Pathogenicity of influenza viruses with genes from the 1918 pandemic virus: functional roles of alveolar macrophages and neutrophils in limiting virus replication and mortality in mice. J Virol (2005) 79(23):14933–44. 10.1128/JVI.79.23.14933-14944.2005 16282492 PMC1287592

[4] Kobasa D Jones SM Shinya K Kash JC Copps J Ebihara H Aberrant innate immune response in lethal infection of macaques with the 1918 influenza virus. Nature (2007) 445(7125):319–23. 10.1038/nature05495 17230189

[5] Brandes M Klauschen F Kuchen S Germain RN. A systems analysis identifies a feedforward inflammatory circuit leading to lethal influenza infection. Cell (2013) 154(1):197–212. 10.1016/j.cell.2013.06.013 23827683 PMC3763506

[6] Garcia-Sastre A. Induction and evasion of type I interferon responses by influenza viruses. Virus Res (2011) 162(1–2):12–8. 10.1016/j.virusres.2011.10.017 22027189 PMC3640439

[7] Julkunen I Sareneva T Pirhonen J Ronni T Melen K Matikainen S. Molecular pathogenesis of influenza A virus infection and virus-induced regulation of cytokine gene expression. Cytokine Growth Factor Rev (2001) 12(2–3):171–80. 10.1016/S1359-6101(00)00026-5 11325600

[8] Kohlmeier JE Woodland DL. Immunity to respiratory viruses. Annu Rev Immunol (2009) 27:61–82. 10.1146/annurev.immunol.021908.132625 18954284

[9] Akaike T Noguchi Y Ijiri S Setoguchi K Suga M Zheng YM Pathogenesis of influenza virus-induced pneumonia: involvement of both nitric oxide and oxygen radicals. Proc Natl Acad Sci U S A (1996) 93(6):2448–53. 10.1073/pnas.93.6.2448 8637894 PMC39817

[10] Narasaraju T Yang E Samy RP Ng HH Poh WP Liew AA Excessive neutrophils and neutrophil extracellular traps contribute to acute lung injury of influenza pneumonitis. Am J Pathol (2011) 179(1):199–210. 10.1016/j.ajpath.2011.03.013 21703402 PMC3123873

[11] Antonopoulou A Baziaka F Tsaganos T Raftogiannis M Koutoukas P Spyridaki A Role of tumor necrosis factor gene single nucleotide polymorphisms in the natural course of 2009 influenza A H1N1 virus infection. Int J Infect Dis (2012) 16(3):e204–8. 10.1016/j.ijid.2011.11.012 22269998

[12] Aranda-Romo S Garcia-Sepulveda CA Comas-Garcia A Lovato-Salas F Salgado-Bustamante M Gomez-Gomez A Killer-cell immunoglobulin-like receptors (KIR) in severe A (H1N1) 2009 influenza infections. Immunogenetics (2012) 64(9):653–62. 10.1007/s00251-012-0623-3 22652695

[13] Esposito S Molteni CG Giliani S Mazza C Scala A Tagliaferri L Toll-like receptor 3 gene polymorphisms and severity of pandemic A/H1N1/2009 influenza in otherwise healthy children. Virol J (2012) 9:270. 10.1186/1743-422X-9-270 23151015 PMC3511245

[14] Zhang YH Zhao Y Li N Peng YC Giannoulatou E Jin RH Interferon-induced transmembrane protein-3 genetic variant rs12252-C is associated with severe influenza in Chinese individuals. Nat Commun (2013) 4:1418. 10.1038/ncomms2433 23361009 PMC3562464

[15] To KK Zhou J Chan JF Yuen KY. Host genes and influenza pathogenesis in humans: an emerging paradigm. Curr Opin Virol (2015) 14:7–15. 10.1016/j.coviro.2015.04.010 26079652 PMC7102748

[16] Sasaki T Irie-Sasaki J Jones RG Oliveira-dos-Santos AJ Stanford WL Bolon B Function of PI3Kgamma in thymocyte development, T cell activation, and neutrophil migration. Science (2000) 287(5455):1040–6. 10.1126/science.287.5455.1040 10669416

[17] Schwindinger WF Robishaw JD. Heterotrimeric G-protein betagamma-dimers in growth and differentiation. Oncogene (2001) 20(13):1653–60. 10.1038/sj.onc.1204181 11313913

[18] Ruckle T Schwarz MK Rommel C. PI3Kgamma inhibition: towards an ‘aspirin of the 21st century’? Nat Rev Drug Discov (2006) 5(11):903–18. 10.1038/nrd2145 17080027

[19] Condliffe AM Cadwallader KA Walker TR Rintoul RC Cowburn AS Chilvers ER. Phosphoinositide 3-kinase: a critical signalling event in pulmonary cells. Respir Res (2000) 1(1):24–9. 10.1186/rr8 11667961 PMC59538

[20] Guillermet-Guibert J Bjorklof K Salpekar A Gonella C Ramadani F Bilancio A The p110beta isoform of phosphoinositide 3-kinase signals downstream of G protein-coupled receptors and is functionally redundant with p110gamma. Proc Natl Acad Sci U S A (2008) 105(24):8292–7. 10.1073/pnas.0707761105 18544649 PMC2448830

[21] Ehrhardt C Marjuki H Wolff T Nurnberg B Planz O Pleschka S Bivalent role of the phosphatidylinositol-3-kinase (PI3K) during influenza virus infection and host cell defence. Cell Microbiol (2006) 8(8):1336–48. 10.1111/j.1462-5822.2006.00713.x 16882036

[22] Ehrhardt C Wolff T Pleschka S Planz O Beermann W Bode JG Influenza A virus NS1 protein activates the PI3K/Akt pathway to mediate antiapoptotic signaling responses. J Virol (2007) 81(7):3058–67. 10.1128/JVI.02082-06 17229704 PMC1866065

[23] Guillot L Le Goffic R Bloch S Escriou N Akira S Chignard M Involvement of toll-like receptor 3 in the immune response of lung epithelial cells to double-stranded RNA and influenza A virus. J Biol Chem (2005) 280(7):5571–80. 10.1074/jbc.M410592200 15579900

[24] Nobs SP Schneider C Heer AK Huotari J Helenius A Kopf M PI3Kgamma is critical for dendritic cell-mediated CD8+ T cell priming and viral clearance during influenza virus infection. PLoS Pathog (2016) 12(3):e1005508 10.1371/journal.ppat.1005508 27030971 PMC4816423

[25] Garcia CC Russo RC Guabiraba R Fagundes CT Polidoro RB Tavares LP Platelet-activating factor receptor plays a role in lung injury and death caused by influenza A in mice. PLoS Pathog (2010) 6(11):e1001171. 10.1371/journal.ppat.1001171 21079759 PMC2974216

[26] Barbosa RP Salgado AP Garcia CC Filho BG Goncalves AP Lima BH Protective immunity and safety of a genetically modified influenza virus vaccine. PLoS One (2014) 9(6):e98685. 10.1371/journal.pone.0098685 24927156 PMC4057169

[27] Russo RC Guabiraba R Garcia CC Barcelos LS Roffe E Souza AL Role of the chemokine receptor CXCR2 in bleomycin-induced pulmonary inflammation and fibrosis. Am J Respir Cell Mol Biol (2009) 40(4):410–21. 10.1165/rcmb.2007-0364OC 18836137

[28] Russo RC Garcia CC Barcelos LS Rachid MA Guabiraba R Roffe E Phosphoinositide 3-kinase gamma plays a critical role in bleomycin-induced pulmonary inflammation and fibrosis in mice. J Leukoc Biol (2011) 89(2):269–82. 10.1189/jlb.0610346 21048214

[29] Garcia CC Weston-Davies W Russo RC Tavares LP Rachid MA Alves-Filho JC Complement C5 activation during influenza A infection in mice contributes to neutrophil recruitment and lung injury. PLoS One (2013) 8(5):e64443. 10.1371/journal.pone.0064443 23696894 PMC3655967

[30] Livak KJ Schmittgen TD. Analysis of relative gene expression data using real-time quantitative PCR and the 2(-Delta Delta C(T)) Method. Methods (2001) 25(4):402–8. 10.1006/meth.2001.1262 11846609

[31] Kehdy FS Gouveia MH Machado M Magalhaes WC Horimoto AR Horta BL Origin and dynamics of admixture in Brazilians and its effect on the pattern of deleterious mutations. Proc Natl Acad Sci U S A (2015) 112(28):8696–701. 10.1073/pnas.1504447112 26124090 PMC4507185

[32] Auton A Brooks LD Durbin RM Garrison EP Kang HM Korbel JO A global reference for human genetic variation. Nature (2015) 526(7571):68–74. 10.1038/nature15393 26432245 PMC4750478

[33] Hrincius ER Dierkes R Anhlan D Wixler V Ludwig S Ehrhardt C. Phosphatidylinositol-3-kinase (PI3K) is activated by influenza virus vRNA via the pathogen pattern receptor Rig-I to promote efficient type I interferon production. Cell Microbiol (2011) 13(12):1907–19. 10.1111/j.1462-5822.2011.01680.x 21899695

[34] Dickensheets H Sheikh F Park O Gao B Donnelly RP Interferon-lambda (IFN-lambda) induces signal transduction and gene expression in human hepatocytes, but not in lymphocytes or monocytes. J Leukoc Biol (2013) 93(3):377–85. 10.1189/jlb.0812395 23258595 PMC3579021

[35] Di Domizio J Blum A Gallagher-Gambarelli M Molens JP Chaperot L Plumas J. TLR7 stimulation in human plasmacytoid dendritic cells leads to the induction of early IFN-inducible genes in the absence of type I IFN. Blood (2009) 114(9):1794–802. 10.1182/blood-2009-04-216770 19553637 PMC2847942

[36] Schif-Zuck S Gross N Assi S Rostoker R Serhan CN Ariel A. Saturated-efferocytosis generates pro-resolving CD11b low macrophages: modulation by resolvins and glucocorticoids. Eur J Immunol (2011) 41(2):366–79. 10.1002/eji.201040801 21268007 PMC3082320

[37] Ariel A Serhan CN. New lives given by cell death: macrophage differentiation following their encounter with apoptotic leukocytes during the resolution of inflammation. Front Immunol (2012) 3:4. 10.3389/fimmu.2012.00004 22566890 PMC3342362

[38] Vago JP Tavares LP Garcia CC Lima KM Perucci LO Vieira EL The role and effects of glucocorticoid-induced leucine zipper in the context of inflammation resolution. J Immunol (2015) 194(10):4940–50. 10.4049/jimmunol.1401722 25876761

[39] Sugimoto MA Ribeiro ALC Costa BRC Vago JP Lima KM Carneiro FS Plasmin and plasminogen induce macrophage reprogramming and regulate key steps of inflammation resolution via annexin A1. Blood (2017) 129(21):2896–907. 10.1182/blood-2016-09-742825 28320709 PMC5445571

[40] Dalli J Serhan CN. Pro-resolving mediators in regulating and conferring macrophage function. Front Immunol (2017) 8:1400. 10.3389/fimmu.2017.01400 29163481 PMC5671941

[41] Pinho V Russo RC Amaral FA de Sousa LP Barsante MM de Souza DG Tissue- and stimulus-dependent role of phosphatidylinositol 3-kinase isoforms for neutrophil recruitment induced by chemoattractants in vivo. J Immunol (2007) 179(11):7891–8. 10.4049/jimmunol.179.11.7891 18025236

[42] Nathan C Shiloh MU. Reactive oxygen and nitrogen intermediates in the relationship between mammalian hosts and microbial pathogens. Proc Natl Acad Sci U S A (2000) 97(16):8841–8. 10.1073/pnas.97.16.8841 10922044 PMC34021

[43] Castor MG Rezende BM Bernardes PT Vieira AT Vieira EL Arantes RM PI3Kgamma controls leukocyte recruitment, tissue injury, and lethality in a model of graft-versus-host disease in mice. J Leukoc Biol (2011) 89(6):955–64. 10.1189/jlb.0810464 21402770

[44] Yum HK Arcaroli J Kupfner J Shenkar R Penninger JM Sasaki T Involvement of phosphoinositide 3-kinases in neutrophil activation and the development of acute lung injury. J Immunol (2001) 167(11):6601–8. 10.4049/jimmunol.167.11.6601 11714830

[45] Hirsch E Katanaev VL Garlanda C Azzolino O Pirola L Silengo L Central role for G protein-coupled phosphoinositide 3-kinase gamma in inflammation. Science (2000) 287(5455):1049–53. 10.1126/science.287.5455.1049 10669418

[46] Maus UA Backi M Winter C Srivastava M Schwarz MK Ruckle T Importance of phosphoinositide 3-kinase gamma in the host defense against pneumococcal infection. Am J Respir Crit Care Med (2007) 175(9):958–66. 10.1164/rccm.200610-1533OC 17322108

[47] Martin D Galisteo R Molinolo AA Wetzker R Hirsch E Gutkind JS PI3Kgamma mediates kaposi’s sarcoma-associated herpesvirus vGPCR-induced sarcomagenesis. Cancer Cell (2011) 19(6):805–13. 10.1016/j.ccr.2011.05.005 21665152 PMC3170773

[48] Conte E Gili E Fruciano M Korfei M Fagone E Iemmolo M PI3K p110gamma overexpression in idiopathic pulmonary fibrosis lung tissue and fibroblast cells: in vitro effects of its inhibition. Lab Invest (2013) 93(5):566–76. 10.1038/labinvest.2013.6 23439433

[49] Shin YK Liu Q Tikoo SK Babiuk LA Zhou Y. Effect of the phosphatidylinositol 3-kinase/Akt pathway on influenza A virus propagation. J Gen Virol (2007) 88(Pt 3):942–50. 10.1099/vir.0.82483-0 17325368

[50] Li W Wang G Zhang H Shen Y Dai J Wu L Inability of NS1 protein from an H5N1 influenza virus to activate PI3K/Akt signaling pathway correlates to the enhanced virus replication upon PI3K inhibition. Vet Res (2012) 43(1):36. 10.1186/1297-9716-43-36 22530768 PMC3416684

[51] Seo SU Kwon HJ Ko HJ Byun YH Seong BL Uematsu S Type I interferon signaling regulates Ly6C(hi) monocytes and neutrophils during acute viral pneumonia in mice. PLoS Pathog (2011) 7(2):e1001304. 10.1371/journal.ppat.1001304 21383977 PMC3044702

[52] Hui KP Lee SM Cheung CY Ng IH Poon LL Guan Y Induction of proinflammatory cytokines in primary human macrophages by influenza A virus (H5N1) is selectively regulated by IFN regulatory factor 3 and p38 MAPK. J Immunol (2009) 182(2):1088–98. 10.4049/jimmunol.182.2.1088 19124752

[53] Borgeling Y Schmolke M Viemann D Nordhoff C Roth J Ludwig S. Inhibition of p38 mitogen-activated protein kinase impairs influenza virus-induced primary and secondary host gene responses and protects mice from lethal H5N1 infection. J Biol Chem (2014) 289(1):13–27. 10.1074/jbc.M113.469239 24189062 PMC3879537

[54] Kaur S Sassano A Joseph AM Majchrzak-Kita B Eklund EA Verma A Dual regulatory roles of phosphatidylinositol 3-kinase in IFN signaling. J Immunol (2008) 181(10):7316–23. 10.4049/jimmunol.181.10.7316 18981154 PMC2597572

[55] Chen-Yu Hsu A Starkey MR Hanish I Parsons K Haw TJ Howland LJ Targeting PI3K-p110alpha suppresses influenza virus infection in chronic obstructive pulmonary disease. Am J Respir Crit Care Med (2015) 191(9):1012–23. 10.1164/rccm.201501-0188OC 25751541

[56] Yang KY Arcaroli J Kupfner J Pitts TM Park JS Strasshiem D Involvement of phosphatidylinositol 3-kinase gamma in neutrophil apoptosis. Cell Signal (2003) 15(2):225–33. 10.1016/S0898-6568(02)00063-3 12464394

[57] Mandelboim O Lieberman N Lev M Paul L Arnon TI Bushkin Y Recognition of haemagglutinins on virus-infected cells by NKp46 activates lysis by human NK cells. Nature (2001) 409(6823):1055–60. 10.1038/35059110 11234016

[58] Lawrence CW Braciale TJ. Activation, differentiation, and migration of naive virus-specific CD8+ T cells during pulmonary influenza virus infection. J Immunol (2004) 173(2):1209–18. 10.4049/jimmunol.173.2.1209 15240712

[59] Wareing MD Lyon AB Lu B Gerard C Sarawar SR. Chemokine expression during the development and resolution of a pulmonary leukocyte response to influenza A virus infection in mice. J Leukoc Biol (2004) 76(4):886–95. 10.1189/jlb.1203644 15240757

[60] Lim K Hyun YM Lambert-Emo K Capece T Bae S Miller R Neutrophil trails guide influenza-specific CD8(+) T cells in the airways. Science (2015) 349(6252):aaa4352 10.1126/science.aaa4352 26339033 PMC4809646

[61] Reif K Okkenhaug K Sasaki T Penninger JM Vanhaesebroeck B Cyster JG. Cutting edge: differential roles for phosphoinositide 3-kinases, p110gamma and p110delta, in lymphocyte chemotaxis and homing. J Immunol (2004) 173(4):2236–40. 10.4049/jimmunol.173.4.2236 15294934

[62] Rodriguez-Borlado L Barber DF Hernandez C Rodriguez-Marcos MA Sanchez A Hirsch E Phosphatidylinositol 3-kinase regulates the CD4/CD8 T cell differentiation ratio. J Immunol (2003) 170(9):4475–82. 10.4049/jimmunol.170.9.4475 12707323

[63] Imai Y Kuba K Neely GG Yaghubian-Malhami R Perkmann T van Loo G Identification of oxidative stress and toll-like receptor 4 signaling as a key pathway of acute lung injury. Cell (2008) 133(2):235–49. 10.1016/j.cell.2008.02.043 18423196 PMC7112336

[64] Vlahos R Stambas J Bozinovski S Broughton BR Drummond GR Selemidis S. Inhibition of Nox2 oxidase activity ameliorates influenza A virus-induced lung inflammation. PLoS Pathog (2011) 7(2):e1001271. 10.1371/journal.ppat.1001271 21304882 PMC3033375

[65] Lehmann K Muller JP Schlott B Skroblin P Barz D Norgauer J PI3Kgamma controls oxidative bursts in neutrophils via interactions with PKCalpha and p47phox. Biochem J (2009) 419(3):603–10. 10.1042/BJ20081268 18983267

[66] Condliffe AM Davidson K Anderson KE Ellson CD Crabbe T Okkenhaug K Sequential activation of class IB and class IA PI3K is important for the primed respiratory burst of human but not murine neutrophils. Blood (2005) 106(4):1432–40. 10.1182/blood-2005-03-0944 15878979

[67] Nigorikawa K Hazeki K Kumazawa T Itoh Y Hoshi M Hazeki O Class-IA phosphoinositide 3-kinase p110beta triggers GPCR-induced superoxide production in p110gamma-deficient murine neutrophils. J Pharmacol Sci (2012) 120(4):270–9. 10.1254/jphs.12134FP 23149576

[68] Fumagalli L Campa CC Germena G Lowell CA Hirsch E Berton G. Class I phosphoinositide-3-kinases and SRC kinases play a nonredundant role in regulation of adhesion-independent and -dependent neutrophil reactive oxygen species generation. J Immunol (2013) 190(7):3648–60. 10.4049/jimmunol.1201951 23447687 PMC4280093

[69] Park HS Jung HY Park EY Kim J Lee WJ Bae YS. Cutting edge: direct interaction of TLR4 with NAD(P)H oxidase 4 isozyme is essential for lipopolysaccharide-induced production of reactive oxygen species and activation of NF-kappa B. J Immunol (2004) 173(6):3589–93. 10.4049/jimmunol.173.6.3589 15356101

[70] Dewas C Dang PM Gougerot-Pocidalo MA El-Benna J. TNF-alpha induces phosphorylation of p47(phox) in human neutrophils: partial phosphorylation of p47phox is a common event of priming of human neutrophils by TNF-alpha and granulocyte-macrophage colony-stimulating factor. J Immunol (2003) 171(8):4392–8. 10.4049/jimmunol.171.8.4392 14530365

[71] Lazrak A Iles KE Liu G Noah DL Noah JW Matalon S. Influenza virus M2 protein inhibits epithelial sodium channels by increasing reactive oxygen species. FASEB J (2009) 23(11):3829–42. 10.1096/fj.09-135590 19596899 PMC2775009

[72] Wong SS Oshansky CM Guo XJ Ralston J Wood T Seeds R Severe influenza is characterized by prolonged immune activation: results from the SHIVERS Cohort Study. J Infect Dis (2018) 217(2):245–56. 10.1093/infdis/jix571 29112724 PMC7335675

[73] Walker EH Perisic O Ried C Stephens L Williams RL. Structural insights into phosphoinositide 3-kinase catalysis and signalling. Nature (1999) 402(6759):313–20. 10.1038/46319 10580505

[74] Wain LV Verwoert GC O’Reilly PF Shi G Johnson T Johnson AD Genome-wide association study identifies six new loci influencing pulse pressure and mean arterial pressure. Nat Genet (2011) 43(10):1005–11. 10.1038/ng.922 21909110 PMC3445021

[75] Adams JN Raffield LM Freedman BI Langefeld CD Ng MC Carr JJ Analysis of common and coding variants with cardiovascular disease in the Diabetes Heart Study. Cardiovasc Diabetol (2014) 13:77. 10.1186/1475-2840-13-77 24725463 PMC4021556

[76] Sawczuk M Maciejewska-Karlowska A Skotarczak B Pawlik A Association between single nucleotide polymorphism rs342286 near the PIK3CG gene and acute coronary syndromes. Pol Arch Med Wewn (2014) 124(4):210–2. 10.20452/pamw.2198 24556817

[77] Johnson AD Yanek LR Chen MH Faraday N Larson MG Tofler G Genome-wide meta-analyses identifies seven loci associated with platelet aggregation in response to agonists. Nat Genet (2010) 42(7):608–13. 10.1038/ng.604 20526338 PMC3057573

[78] Kachele M Hennige AM Machann J Hieronimus A Lamprinou A Machicao F Variation in the phosphoinositide 3-kinase gamma gene affects plasma HDL-cholesterol without modification of metabolic or inflammatory markers. PLoS One (2015) 10(12):e0144494. 10.1371/journal.pone.0144494 26658747 PMC4675530

[79] Keynan Y Malik S Fowke KR. The role of polymorphisms in host immune genes in determining the severity of respiratory illness caused by pandemic H1N1 influenza. Public Health Genomics (2013) 16(1–2):9–16. 10.1159/000345937 23548712

[80] Ciancanelli MJ Abel L Zhang SY Casanova JL. Host genetics of severe influenza: from mouse Mx1 to human IRF7. Curr Opin Immunol (2016) 38:109–20. 10.1016/j.coi.2015.12.002 26761402 PMC4733643

