# Supplementary figures and images for: Phosphatidyl Inositol 3 Kinase-Gamma Balances Antiviral and Inflammatory Responses During Influenza A H1N1 Infection: From Murine Model to Genetic Association in Patients
Source: Front Immunol. 2018 May 15;9:975. doi: 10.3389/fimmu.2018.00975 (PMC5962662; doi:10.3389/fimmu.2018.00975)

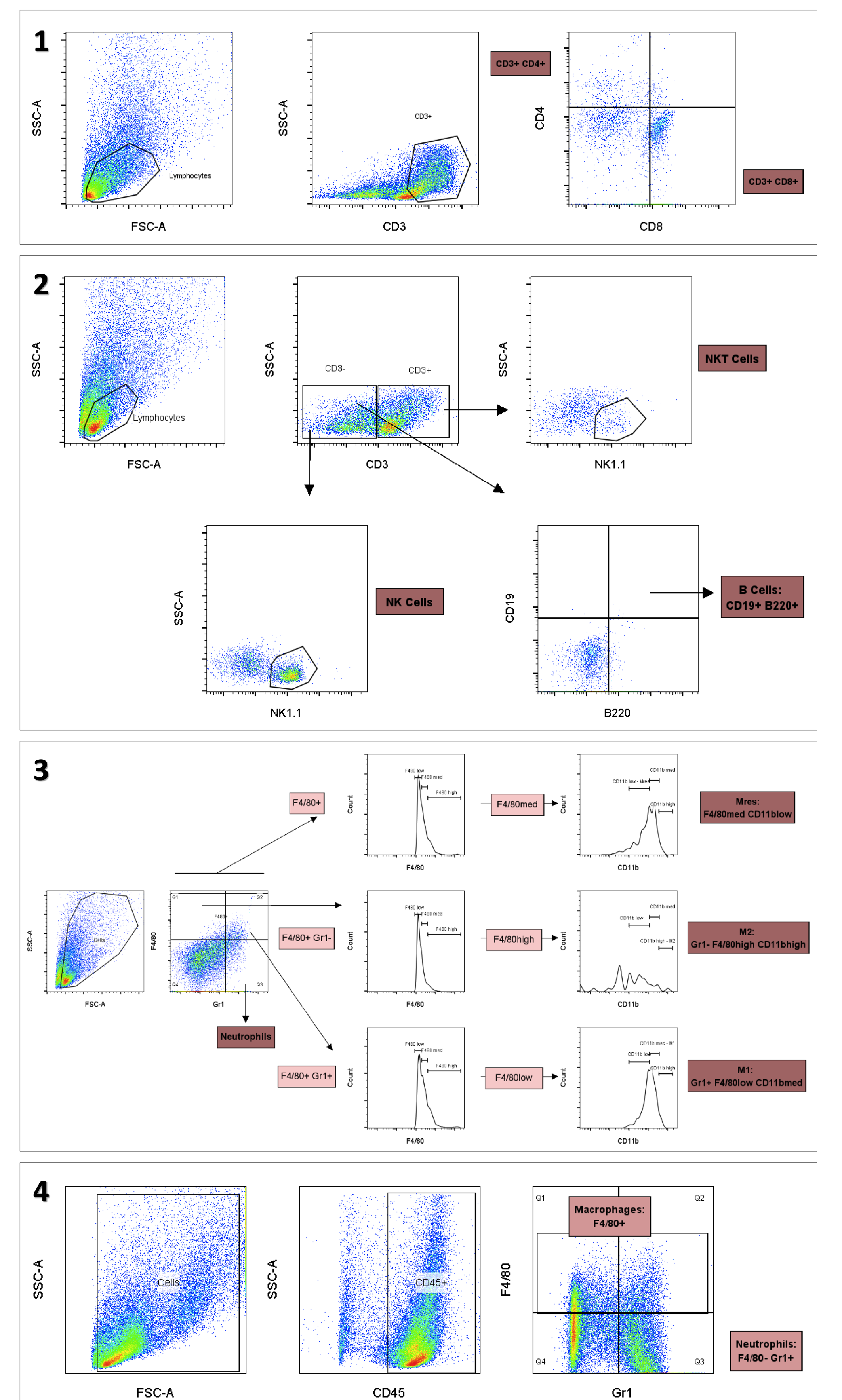

Supplement: Figure S1 — Gating strategies for FACS analysis of lymphocytes (1), natural killer (NK) and B cells (2), macrophages and neutrophils in bronchoalveolar lavage fluid (3), and macrophages and neutrophils in lungs (4). [file image_1.tif]
